# Geophysical characterization of subsurface structures for optimal planning in the Abu Tartur phosphate mine

**DOI:** 10.1038/s41598-026-48186-y

**Published:** 2026-04-21

**Authors:** Gehad M. K. Ahmed, M. M. Senosy, Gamal Y. Boghdady, Mosaad Ali Hussein Ali

**Affiliations:** 1 Department of Mining and Metallurgical Engineering, Faculty of Engineering, Assiut University, Assiut, Egypt; 2 Geology Department, Faculty of Science, Assiut University, Assiut, Egypt

**Keywords:** Abu Tartur mine, Aeromagnetic, Bouguer gravity, Subsurface structures, Geology, Geophysics

## Abstract

Optimal planning of the Abu Tartur Mine (ATPM) in Egypt requires a comprehensive understanding of the region’s phosphate ore geology. The recent closure of the subsurface ATPM was primarily due to insufficient geological data, including unrecognized faults that concealed phosphate beds. The present study integrates gravity and magnetic geophysical methods to characterize the phosphate beds and identify geological structures. Aeromagnetic Reduced to Pole (RTP) and Bouguer anomaly data were used, with filters (analytical signal, first vertical derivative, high-pass and low-pass (applied to enhance interpretation. Through 2D gravity and magnetic modelling, the subsurface sedimentary sequence above the basement rocks was defined. The subsea depth to the subsurface rock layer boundaries was determined. Consequently, structure contour maps were created for the basement and the Nubian sandstone surfaces, along with Isopach map of the phosphatic rocks. Maps and filtered data revealed the predominant subsurface structures controlling the phosphate distribution. These structures are folds (plunging and double-plunging synclines and anticlines with axes trending NE-SW, NNW-SSE, and NW-SE) and faults (normal and strike-slip). Normal faults bound the ATPM plateau with downthrow directions outward. The thickness of phosphatic rocks varies from 0.8 to 32 m. The limited thickness is recorded in the present ATPM location, whereas the maximum thickness is observed at the troughs of the syncline folds located northeast and southwest of the plateau. Therefore, the ATPM location was suboptimal and uneconomical, while the northeast and southwest areas offer more promising targets for phosphate extraction. This misallocation likely contributed to the mine failure.

## Introduction

Optimal mine planning is essential for sustainable mining practices because it reduces the environmental and economic impacts of resource extraction^1–4^. Through effective subsurface characterization and strategic operational design, mine planning minimizes costs and waste, reduces environmental damage, and extends the lifespan of mineral reserves^5^. This approach supports resource conservation and aligns with sustainable development principles^6,7^. Phosphate ore, a non-renewable but essential resource for agriculture and industry, highlights the importance of efficient resource management^8,9^. As the primary component of phosphate fertilizers, it plays a vital role in global food production by improving soil fertility and crop yields^10,11^ Phosphate is also widely used in animal feed, detergents, and various industrial chemicals^11^. Therefore, improving the efficiency of phosphate extraction is essential for addressing global food security while minimizing the environmental impacts of mining^2,12^.

The Abu Tartur Phosphate Mine (ATPM) in Egypt represents one of the world’s important phosphate reserves and plays a significant role in the country’s economic and industrial development^8^. However, ATPM has faced major operational challenges that led to the closure of its underground mining operations^13^. These challenges are largely attributed to limited geological understanding and unrecognized subsurface structures, highlighting the need for improved subsurface characterization to support effective mine planning and sustainable resource extraction^14,15^. The geological complexity of the ATPM region, characterized by concealed faults and variable thickness of phosphate layers, has historically hindered efficient mining operations^13^. Earlier subsurface investigations relied on incomplete datasets, which resulted in suboptimal mine placement and inefficient resource utilization^16^.

Previous studies addressing underground mine closures have mainly focused on geotechnical aspects such as optimizing extraction panel widths^17^, designing support systems^13^, and determining reinforcement strategies^16^. While these studies provide important insights into structural stability, they often overlook the role of comprehensive geological assessment in understanding operational failures. At ATPM, the transition from underground mining to surface mining behind the plateau left considerable phosphate reserves untapped, highlighting a significant research gap: the limited geological characterization of subsurface structures controlling phosphate distribution. Addressing this gap is essential for improving resource utilization while maintaining economic and environmental sustainability.

Geophysical methods are effective non-invasive tools for subsurface exploration and mineral deposit characterization^18–21^. These methods use variations in the physical properties of rocks to investigate subsurface features and delineate the extent and quality of mineral deposits before mining begins^22–24^. Among these techniques, magnetic methods are widely used for detecting and mapping subsurface anomalies^25–27^. Magnetic surveys have successfully delineated manganese-rich zones in the Aïn Beida mine in Morocco^28^, while high-resolution aeromagnetic data in Ibadan, Nigeria revealed potential mineral deposits using derivative and Analytic Signal Amplitude techniques^29^. Gravity methods have also been applied in gold exploration in South Africa^30^, ^31^ and in the identification of chromite^32^, ^33^, iron^34^, manganese^35^, copper sulphide ores^36,37^, and coal deposits^38,39^. However, due to the complexity of ore-bearing formations, integrating multiple geophysical techniques is often required^40,41^. For example, the integration of gravity and magnetic data in the Sefwi Belt of Ghana successfully delineated zones of gold mineralization and mapped lithological and structural features^42^. Despite these successes, the integration of Bouguer gravity and aeromagnetic data has not been extensively applied for systematic subsurface characterization in the ATPM region, leaving important uncertainties regarding subsurface structures and their influence on phosphate distribution.

The present study addresses this gap by applying an integrated geophysical approach to characterize subsurface structures in the ATPM region. Reduced to the Pole (RTP) aeromagnetic data and Bouguer gravity anomaly data, combined with advanced filtering techniques, are used to identify and model geological structures controlling phosphate layer distribution. Structure contour and thickness maps derived from 2D modelling provide a clearer understanding of the subsurface environment and help identify optimal zones for future mining operations. This approach improves the understanding of structural controls on phosphate distribution and provides guidance for future mining strategies in ATPM, while offering a methodological framework applicable to similar mining environments with complex subsurface geology.

## Location and geological setting of the study area

### Location of study area

**Fig. 1:**
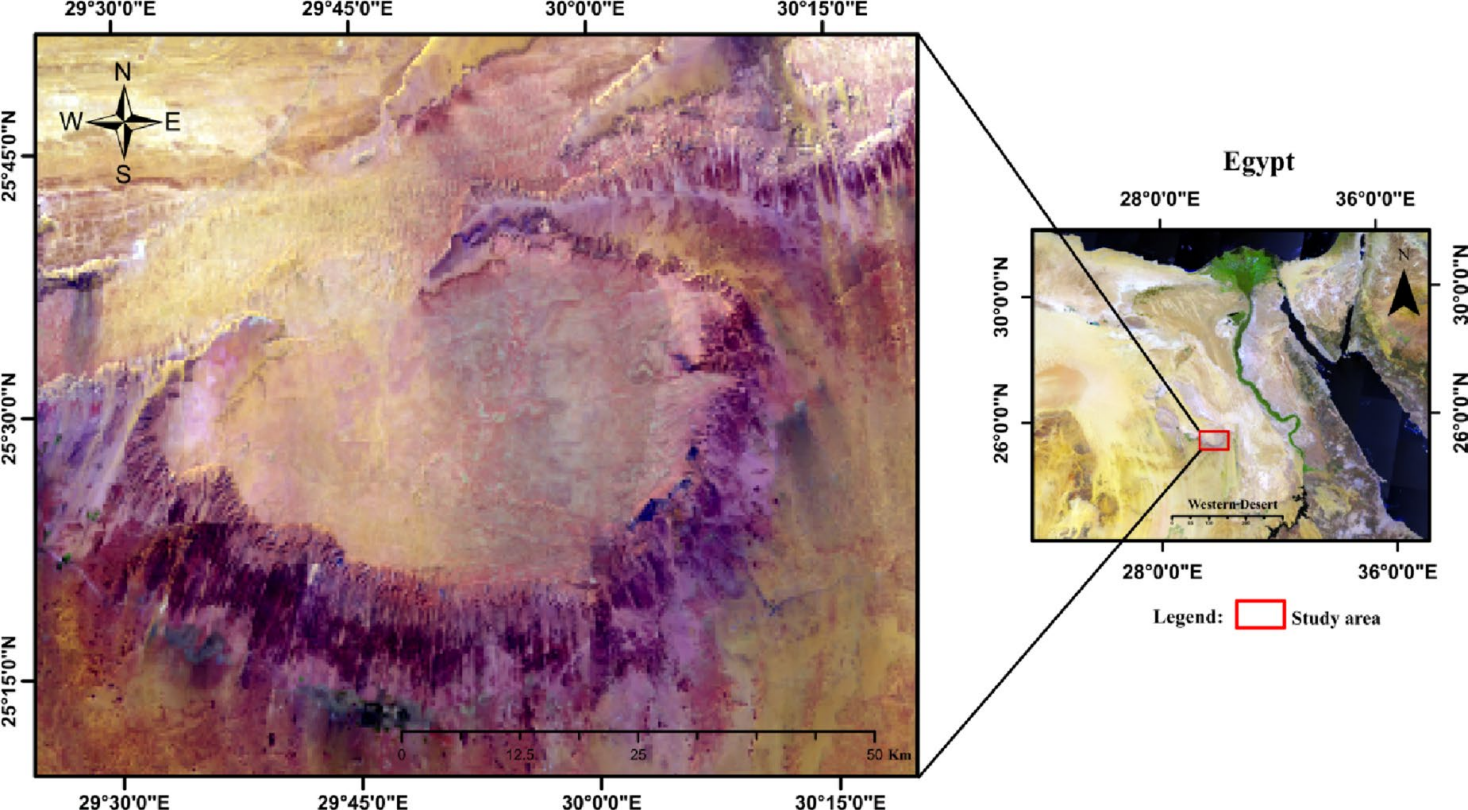
Location map of the study area (generated using ArcMap version 10.8 https://desktop.arcgis.com/en/arcmap/latest/get-started/setup/arcgis-desktop-system-requirements.htm).

Phosphate deposits are distributed in several locations in Egypt along the phosphate belt. The investigated area is part of the Western Desert in Egypt, including the Abu Tartur Plateau, which lie at the western escarpment of the El-Kharga Oasis (Fig. 1). It lies between latitudes 25˚10 `and 25˚50 ` N and longitudes 29˚25 `and 30˚20 ` E.

### General geology

**Fig. 2:**
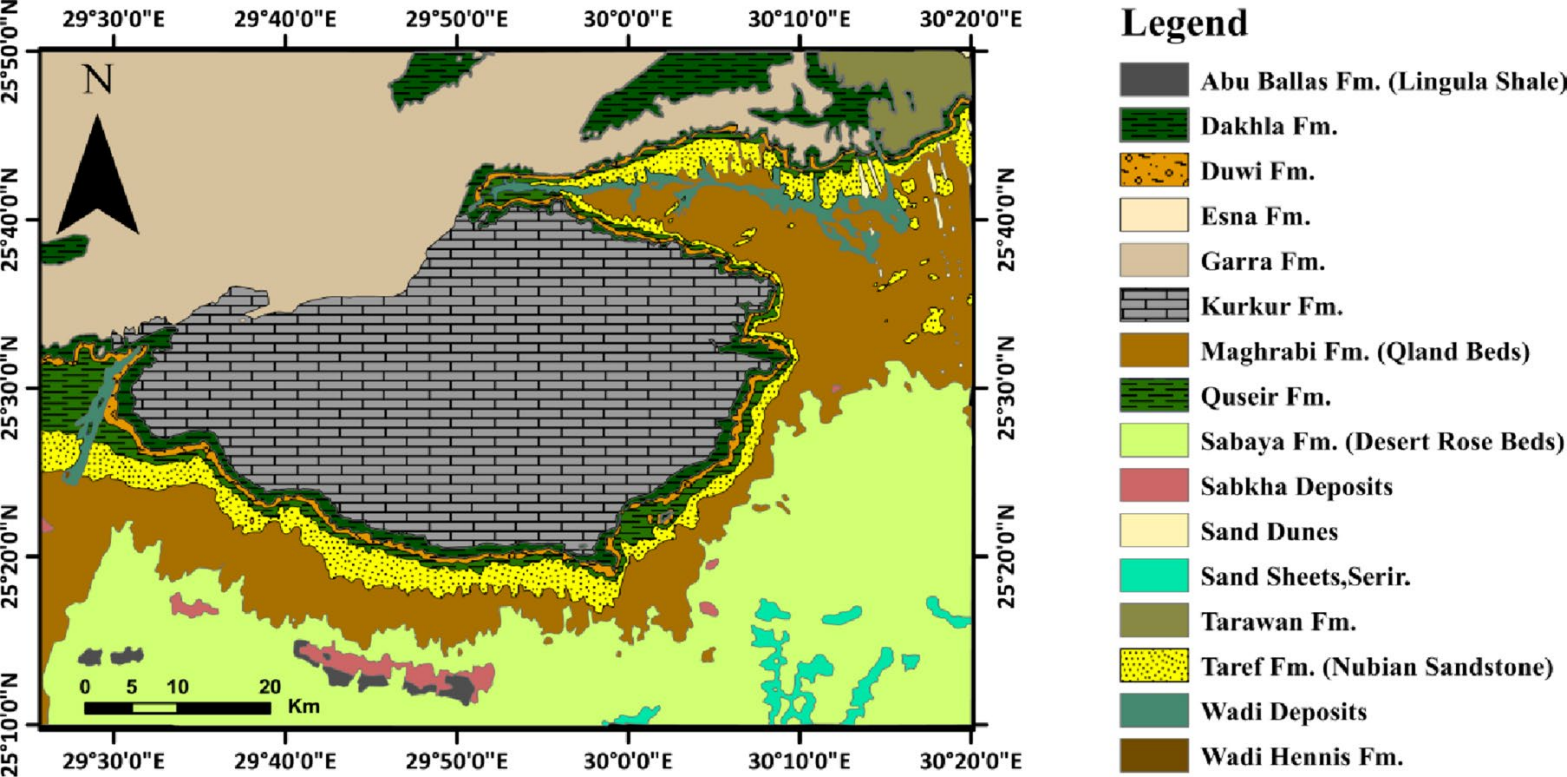
Geological map of the study area (modified after EGPC and Conoco Coral^49^ (generated using ArcMap version 10.8 https://desktop.arcgis.com/en/arcmap/latest/get-started/setup/arcgis-desktop-system-requirements.htm).

According to a geological map covering Egypt’s middle latitudes, phosphorite deposits are primarily found in Hanmraween area (Eastern Desert), the Sebaiya area (along the Nile Valley), and the Western Desert, specifically the Abu Tartur area^43^. Abu Tartur phosphate deposit, which is thought to be the thickest deposit in Egypt, covers an area of roughly 1200 km². The main region is located between El-Kharga Oasis in the east and El-Dakhla Oasis in the west, and includes the Abu Tartur plateau^44^. Most of the sedimentary rocks of the Abu Tartur Plateau region are Pre-Maastrichtian to Quaternary in origin. From oldest to youngest, the Upper Cretaceous Lower Tertiary formations that make up the plateau’s sedimentary sequence, especially on the eastern side (Fig. 2), are the Taref Nubia sandstone formation, Quseir shale formation, Duwi formation (phosphorite), Dakhla shale formation, and Kurkur limestone formation^45^. In Egypt, the Duwi Formation, which consists of phosphatic rocks, is of Late Cretaceous marine transgression and is overlain conformably by the deep-marine shales and marls of the mid-Maastrichtian Dakhla Formation and unconformably overlies the fluvial shale sequence of the mid-Campanian Quseir Formation. The phosphate beds are interbedded with glauconites, argillaceous limestone, and black shale. The economic phosphate bed is at the base of the Duwi formation^46^.

### Structural setting

The Cretaceous and Lower Tertiary depositional environments in southern Egypt, especially the Abu Tartur Plateau, are studied and discussed, based on a combination of paleontological and sedimentological studies, which show that repeated sea level fluctuations and large-scale rejuvenation of pre-existing fault systems determined the Cretaceous to Early Tertiary evolutionary history of the depositional environments of southern Egypt 47. The Dakhla (quite near Abu Tartur) and Assiut Basins formed in Central Egypt in the Late Jurassic or Early Cretaceous, marking the beginning of the progressive subsidence of large intracratonic depressions caused by these fault systems. Sea-level variations and synsedimentary tectonism controlled the transgressions and regressions that filled these depressions with sediments of continental and marine origin. Typical stable-shelf tectonics is responsible for the structural components of the El-Kharga-Dakhla stretch. The basement block movements are indicated by faults and, to a lesser extent, by large gentle folds that are reflected on the surface. The thickness and lithology of the sedimentary cover, especially in its southern regions, most likely determine the nature and severity of the tectonic and deformational events. The predominant tectonic characteristic is faults, which are more persistent and denser. The only area along the El-Kharga-Dakhla stretch where crystalline basement outcrops with a thin sedimentary layer and noticeable basement rock up-arching can be observed is the southern portion of the Kharga Oasis. However, the broad warping and undulations are more noticeable in the west and northwest of El-Kharga, as in the Abu Tartur and El-Dakhla areas 48.

## Methodology and data processing

**Fig. 3:**
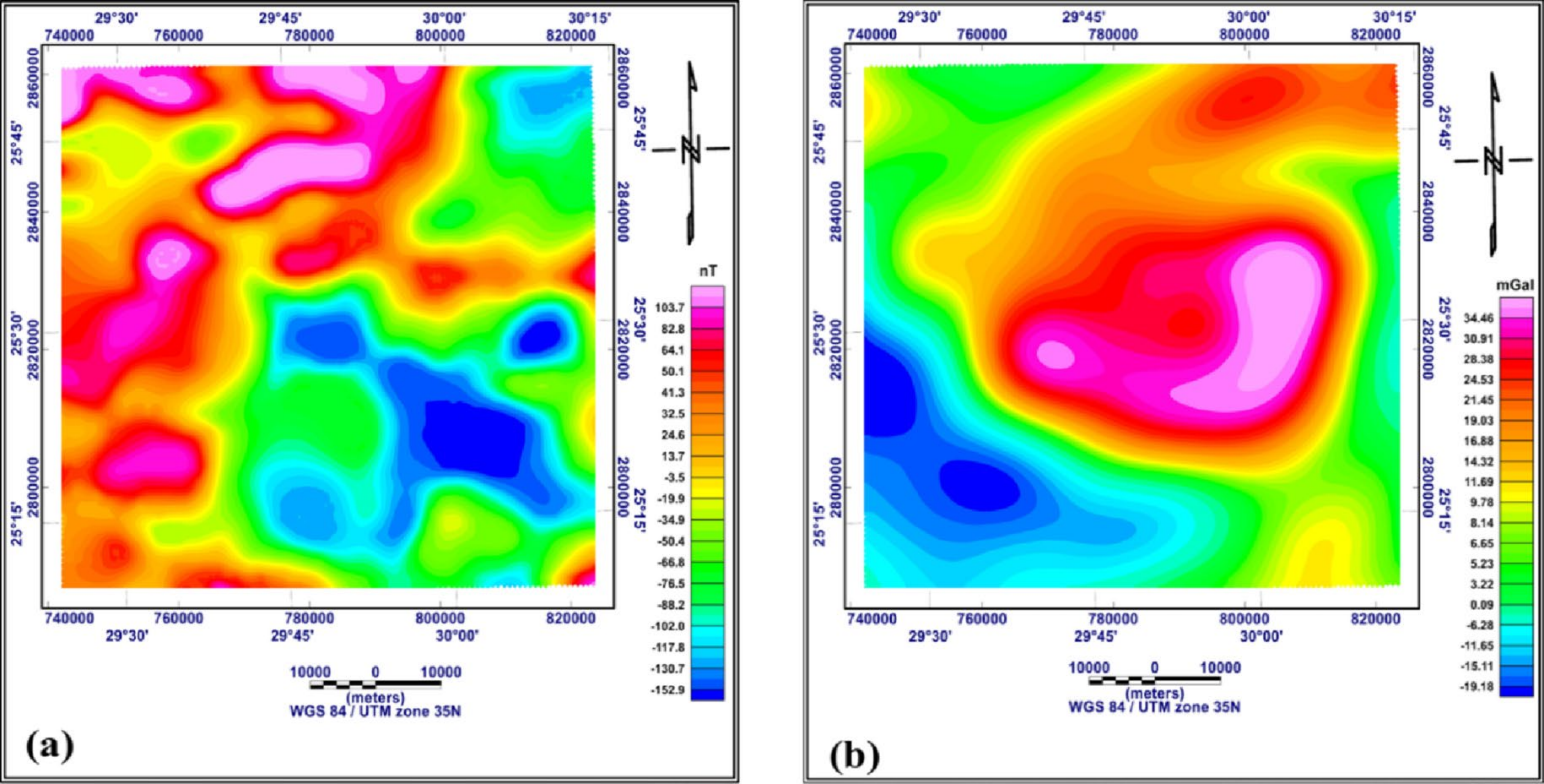
RTP aeromagnetic map of the study area (**a**) Bouguer gravity map (**b**) (generated using Geosoft Oasis Montaj version 8.4 https://community.seequent.com/).

**Fig. 4:**
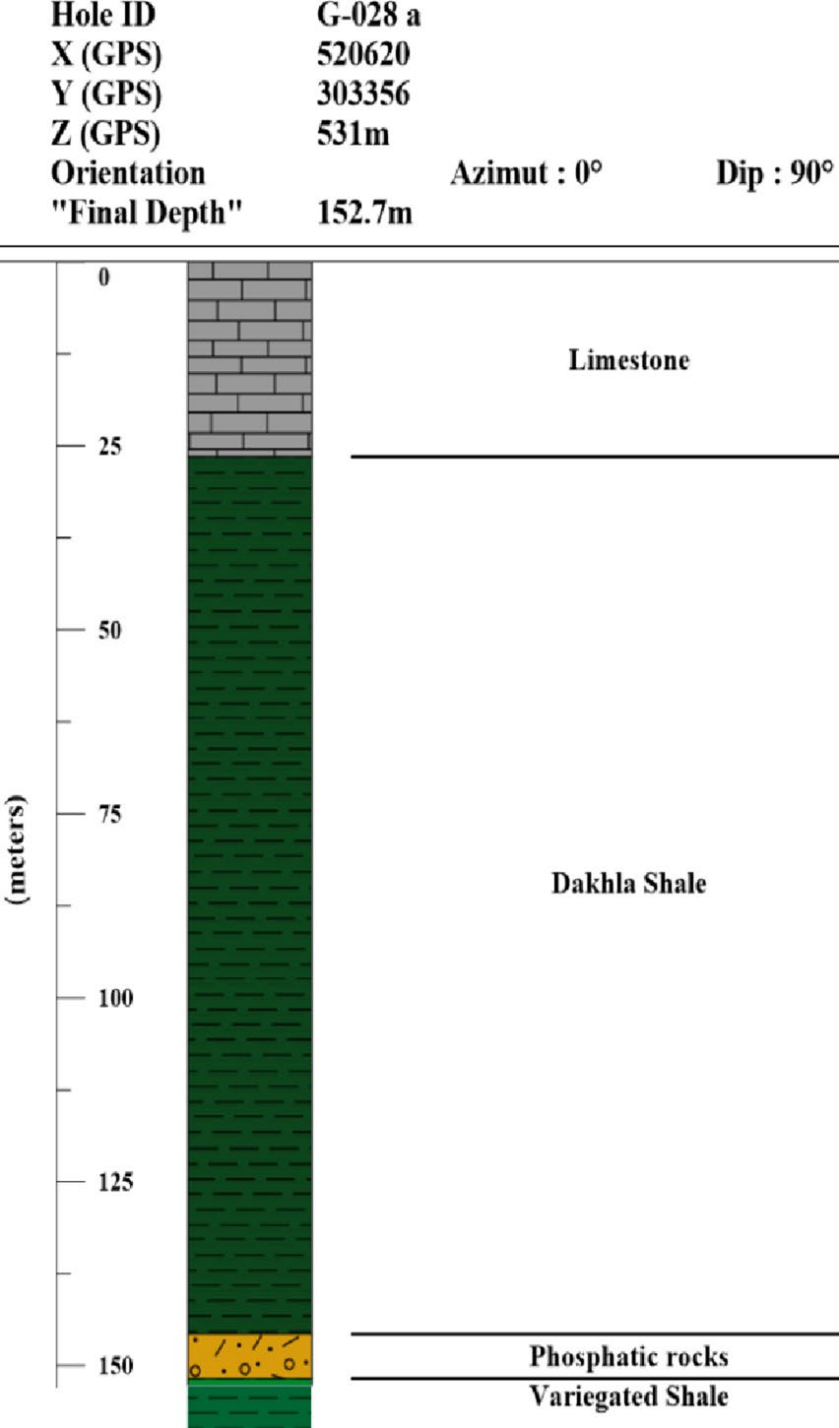
Subsurface lithology of borehole G-028a as an example of boreholes in the study area. (Compiled from Egypt phosphate company) (generated using Strater version 5).

Integration between gravity and magnetic methods is the most effective geophysical technique, which uses disturbances in the Earth’s gravity and magnetic fields to determine the density and magnetic susceptibility of rocks which reflect the subsurface structures. In the present study, gravity and magnetic data were used to provide a general picture of the predominant subsurface structures of the study area. The data were available in the form of reduced to pole (RTP) aeromagnetic data compiled by the Egyptian General Petroleum Corporation EGPC (1989), as shown in Fig. 3a, the line drawn contour interval is 25nT with scale 1:500,000 and the original altitude with terrain clearance equivalent to 1000ft barometric. However, the gravity data in the form of Bouguer gravity data downloaded from the website, https://bgi.obs-mip.fr/, The data were prepared as a contour map, as shown in Fig. 3b. The subsurface lithology was recognised from deep wells in or near the study area, as shown in Fig. 4.

**Fig. 5:**
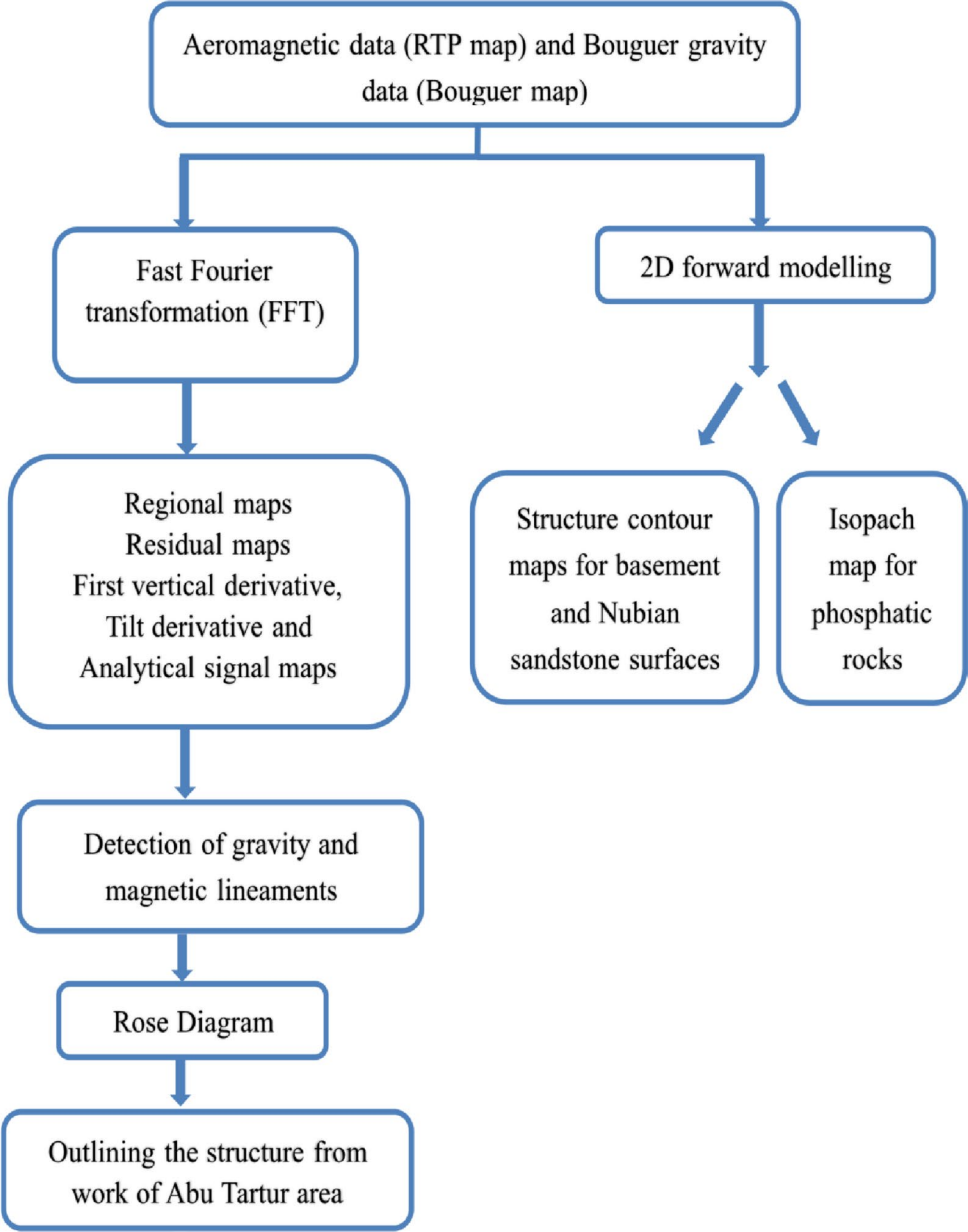
Flowchart illustrating the main processes of analysis that will be performed on the RTP aeromagnetic and Bouguer gravity data of the study area.

Analysis and interpretation of RTP aeromagnetic and Bouguer gravity data were carried out through the following flowchart (Fig. 5).

### Data processing

Both the RTP aeromagnetic and Bouguer gravity data should be filtered to condition the data set, making it easier to interpret the significance of gravity and magnetic anomalies in terms of geological features. In the present study, different filters will be applied on the Bouguer gravity and RTP aeromagnetic data. These filters can summarize as follows:

- The Fast Fourier Transform (FFT) was applied to the data to produce the energy spectrum curve. From the resulting curve, the depths to the shallow and deep sources^50^.
- The (regional) low-pass filter highlights deep, high amplitude sources by suppressing small anomalies and high-frequency noise. As a result, the anomalies appear broader and less sharp than those on the Bouguer anomaly and RTP aeromagnetic maps, while the regional field itself exhibits a smooth overall trend pattern. The (residual) high- pass filter shows the high-frequency and short-wavelength anomalies of limited aerial extension that emphasize the shallowly seated causative bodies^51^,^52^.
- The first vertical derivative filter measures the rate of change of the gravity or magnetic field in the vertical direction. The first vertical derivative is also be positive over the source, zero over the edge, and negative outside a vertical-sided source^53^.
- The tilt derivative filter is a generalized local phase; it was applied to determine the vertical and inclined contacts and/or structure lines and to map shallow basement structures and mineral exploration targets. On the other hand, the tilt derivative exhibits zero crossings near the edges of structures. TDR was estimated by dividing the vertical derivative by the total horizontal derivative, as given in Eq. 1^54,55^.

$$\:TDR=arctan\left(\frac{VDr}{THDR}\right)\:\:\:\:\:\:\:\:\:\:\:\:\:\:\:\:\:\:\:\:\:\:\:\:\:\:\:\:\:\:\:\:\:\left(1\right)$$

- The analytic signal (AS) method, or the total gradient method, is used to define the edges (boundaries) of geologically anomalous magnetization or density distributions 56. The analytic signal technique was discussed elsewhere by ^53,57,58^ as the square root of the sum of the squared vertical and horizontal derivatives of the magnetic field, as given in Eq. 2.

$$\:Asig=\sqrt{d{x}^{2}+d{y}^{2}+d{z}^{2}}\:\:\:\:\:\:\:\:\:\:\:\:\:\:\:\:\:\:\:\:\:\:\:\:\:\left(2\right)$$

All applied filters were carried out by using Geosoft Oasis Montaj version 8.4 ^59^. In addition to the filters applied to the gravity and RTP aeromagnetic data, 2D gravity and magnetic modelling was performed. These models were applied to show the subsurface geologic sequence in the study area along definite directions. The sequence shows the subsurface geologic layers from the surface to the basement rocks. The subsurface structures prevailing in the study area can also be outlined from the model. The modelling process was carried out using an interactive modelling package running on GM-SYS via Geosoft Oasis Montaj version 8.4 ^59^.

## Results and discussion

The qualitative and quantitative interpretation of the Bouguer gravity and Aeromagnetic (RTP) data covering the study area is discussed below.

### Qualitative interpretation

The qualitative interpretation of both gravity and magnetic data includes inspection of the shape, magnitude, amplitude, and linearity of gravity and magnetic anomalies in Bouguer gravity and RTP aeromagnetic maps, as well as first vertical derivative, analytical signal, and tilt derivative maps. The gravity and magnetic lineaments corresponding to linear structures are traced from all the mentioned maps and presented on rose diagrams.

#### RTP aeromagnetic and Bouguer gravity maps

The RTP aeromagnetic map of the study area (Fig. 3a) shows the magnitudes of the magnetic anomalies vary from −152.9 to + 103.7 nT. This great variation in the magnitude of magnetic anomalies indicating that the area is highly affected by different structural sets. The central and southeastern parts of the area show lower magnitudes ranging from − 19.9 to −152.9 nT, indicating that this area is uplifted and that granite basement rocks, which have lower magnetic susceptibility than the surrounding rocks, occur there. The northern part shows a high-magnitude magnetic anomaly, indicating a magnetic source of higher magnetic susceptibility compared with other areas.

The Bouguer gravity map of the study area (Fig. 3b) shows that major parts of the area are covered by positive gravity anomalies which values ranging from 3.22 to 34.46 mGal. The central part of the study area, which includes the Abu Tartur plateau, has the highest positive gravity values. This indicates that the Abu Tartur plateau is an uplifted plateau, whereas the southwestern part, which has negative gravity values ranging from − 6.28 to −19.18 mGal, is downfaulted.

#### Reginal and residual maps of both RTP and Bouguer data

**Fig. 6:**
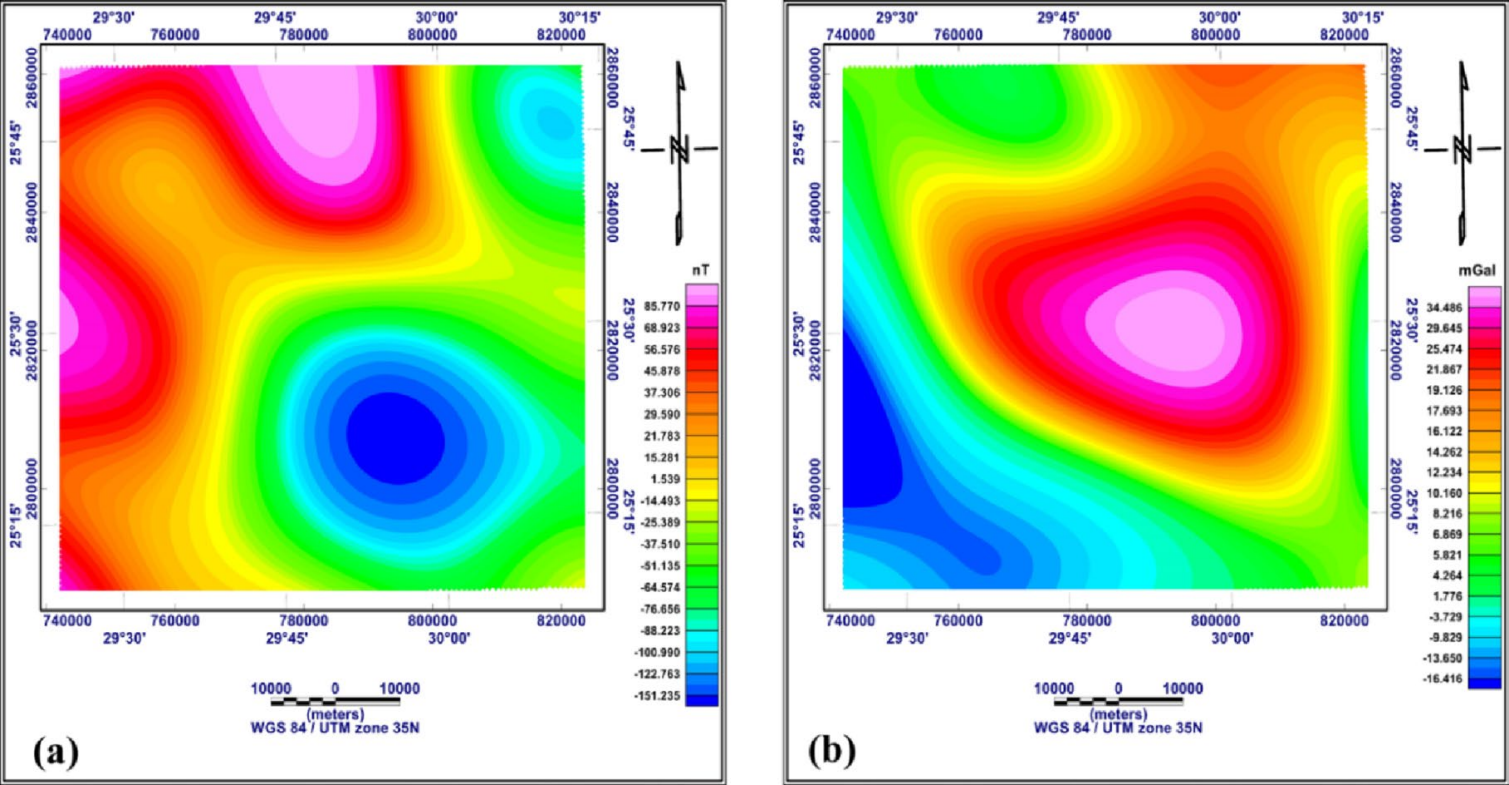
Low pass filter of the RTP aeromagnetic map (**a**) and of the Bouguer gravity map (**b**) (generated using Geosoft Oasis Montaj version 8.4 https://community.seequent.com/).

The high (residual) and low (regional) pass filters were applied with a cutoff of 0.025 cycle/km. The regional magnetic and gravity anomalies are shown in Fig. 6a and b. from these figures, it can be seen that most of the magnetic and gravity anomalies are circular to oval, pointing to circular to subcircular geologic features. The linear regional magnetic and gravity anomalies in W-E and NW-SE directions, indicate deep-seated regional subsurface structures prevailing in both the northern and central parts of the area.

**Fig. 7:**
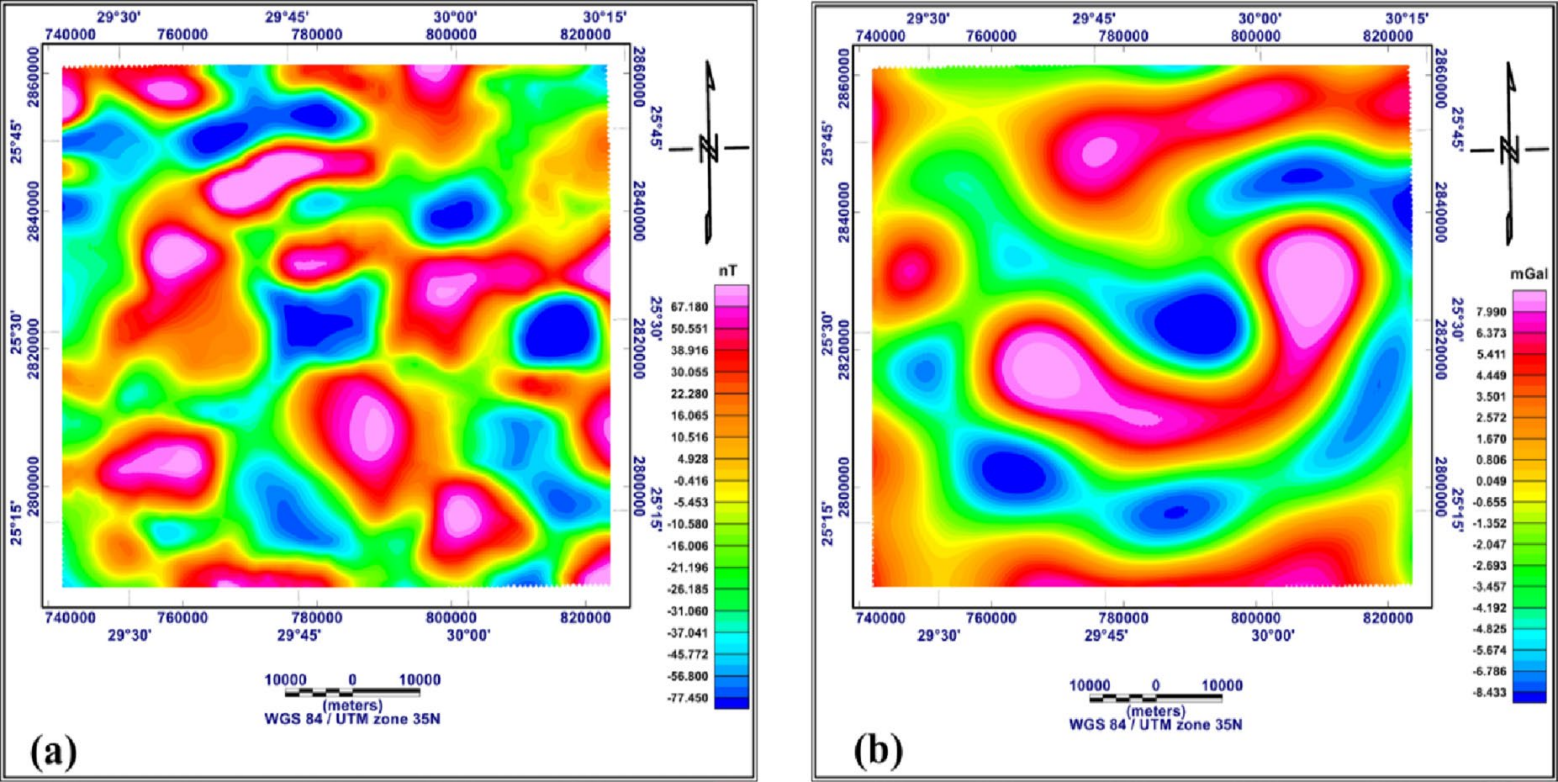
High pass filter of the RTP aeromagnetic map (**a**) and of the Bouguer gravity map (**b**) (generated using Geosoft Oasis Montaj version 8.4 https://community.seequent.com/).

The residual anomaly maps of RTP aeromagnetic and Bouguer gravity are given in Fig. 7a and b. The residual RTP anomalies have positive and negative magnitudes and exhibit circular to semi-circular shapes (Fig. 7a). These anomalies are mostly aligned in W-E and NW-SE directions, indicating shallow geologic features and/or structures extending in these directions. different authors, on the surface, recorded these two structural directions. The Bouguer gravity anomalies (Fig. 7b) show well-defined W-E and WNW-ESE extensions. The W-E extension points to predominant structure lineaments recorded by different authors as W-E strike-slip faults. These fault trends originate in the basement rocks and extend upward into the sedimentary cover.

#### 4.1.3 FVD, TDR, and AS of both RTP aeromagnetic and Bouguer gravity data

**Fig. 8:**
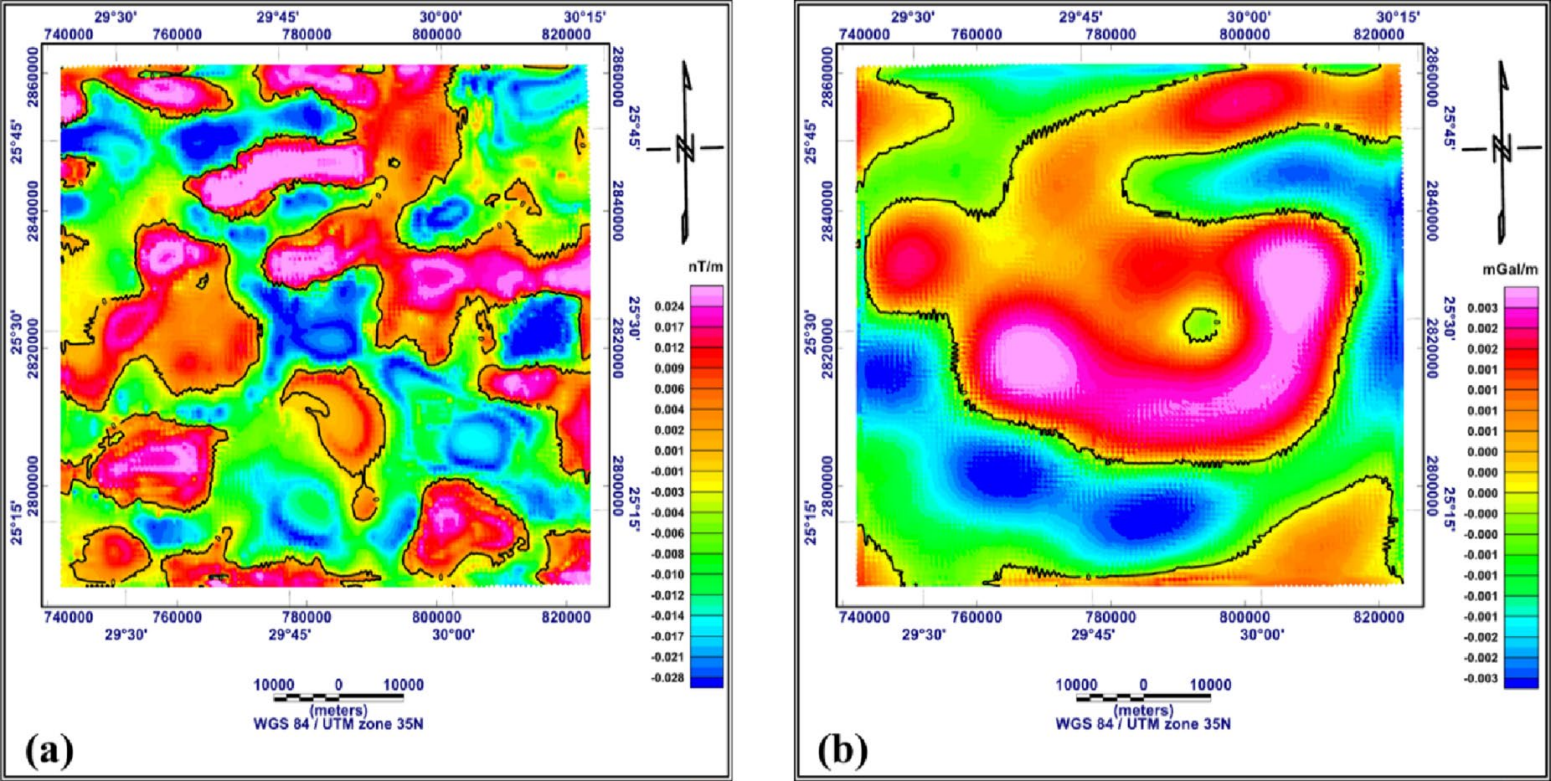
First vertical derivative of the RTP aeromagnetic map (**a**) and of the Bouguer gravity map (**b**), the black lines are the zero contours (generated using Geosoft Oasis Montaj version 8.4 https://community.seequent.com/).

The first vertical derivative (FVD) is an effective tool for enhancing small, weak anomalies that reflect near-surface geologic features and/or structures. Also, it is used to delineate lithologic contacts based on susceptibility and density contrasts (Fig. 8a and b). The first vertical derivative map of the RTP aeromagnetic map (Fig. 8a) shows that, the magnitudes of the magnetic anomalies generally vary from − 0.028 to + 0.024 nT/m. Local anomalies of short wavelengths indicating shallow sources; however, zero contours reflect contacts or faults. From the map, the main directions of the magnetized sources and lithologic as well as structure contacts extend in the NNE-SSW, ENE-WSW, and W-E directions. The first vertical derivative map of the Bouguer gravity data (Fig. 8b) reveals that the anomalies alternate between positive and negative with values reach up to + 0.003 mGal/m and − 0.003 mGal/m, respectively. From the distribution of zero contour lines, the major lithologic contacts and/or structure lines are extending mainly in the ENE-WSW and W-E directions.

**Fig. 9:**
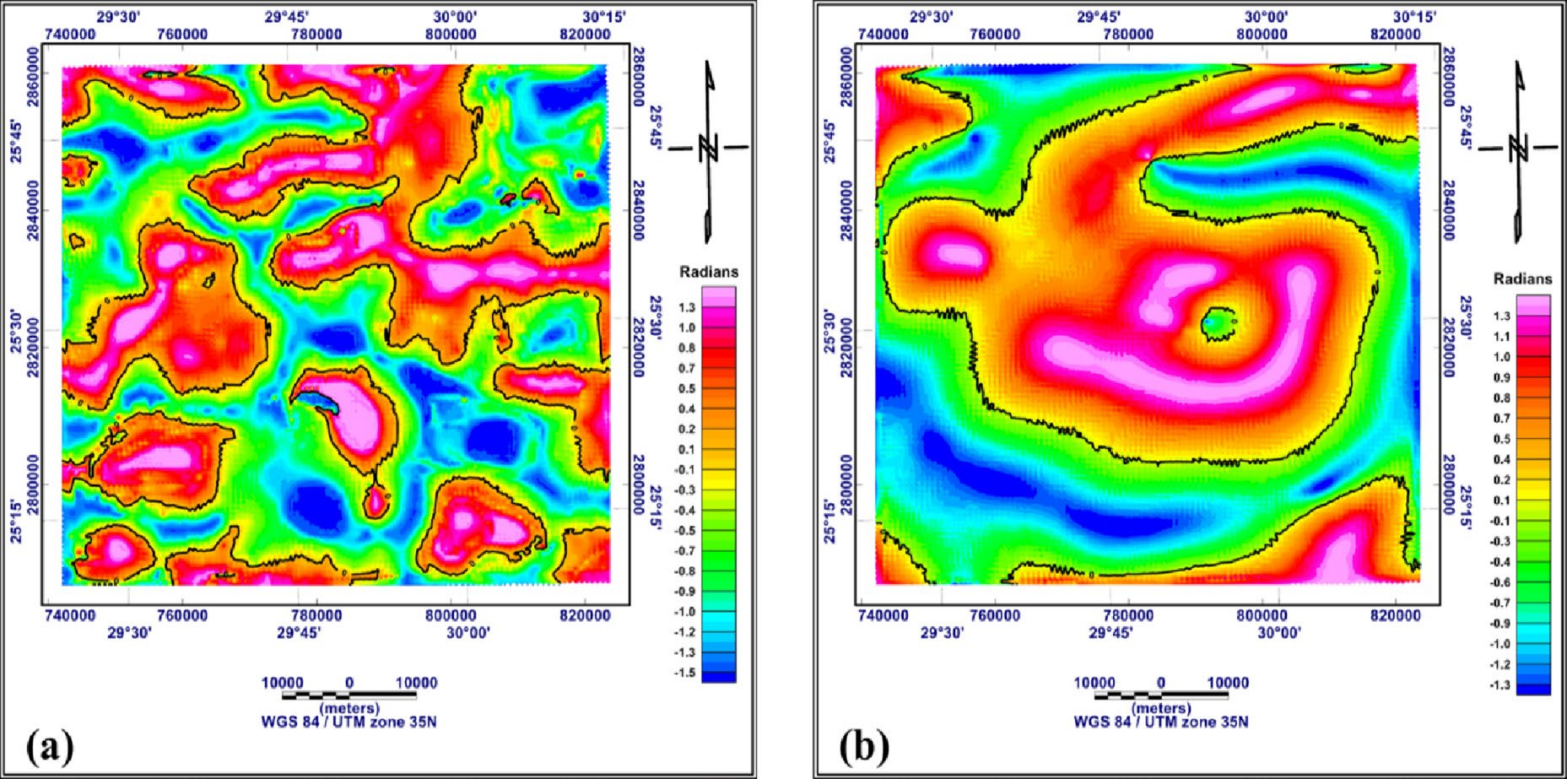
Tilt derivative filter of the RTP aeromagnetic map (**a**) and of the Bouguer gravity map (**b**), the black lines are the zero contours (generated using Geosoft Oasis Montaj version 8.4 https://community.seequent.com/).

The tilt derivative filter (TDR) was applied to both RTP aeromagnetic and Bouguer gravity data to determine the vertical and inclined contacts and/or structure lines. The zero indicate the edges of the causative bodies, which may be structural or lithologic contacts. The resulting maps are shown in Fig. 9a and b, respectively. The TDR map of the RTP aeromagnetic data (Fig. 9a) shows that the anomalies radians range from − 1.5 to + 1.3. The tilt derivative anomalies are similar to the first vertical derivative anomalies in the shape and trends of the major structure. The TDR map of the Bouguer gravity data (Fig. 9b), shows that the TDR values range from − 1.3 and + 1.3 radians. The TDR anomalies of the RTP aeromagnetic data are generally local with W-E extensions; however, the TDR of the Bouguer gravity data consists of three major anomalies extending nearly W-E. The local TDR anomalies in the RTP aeromagnetic data point to local variation in the magnetic susceptibility within the basement rocks; however, the major anomalies in the TDR of the Bouguer gravity data extend in W-E direction, pointing to a major structure that may have originated in the basement rocks and extended upward within the sedimentary cover.

**Fig. 10:**
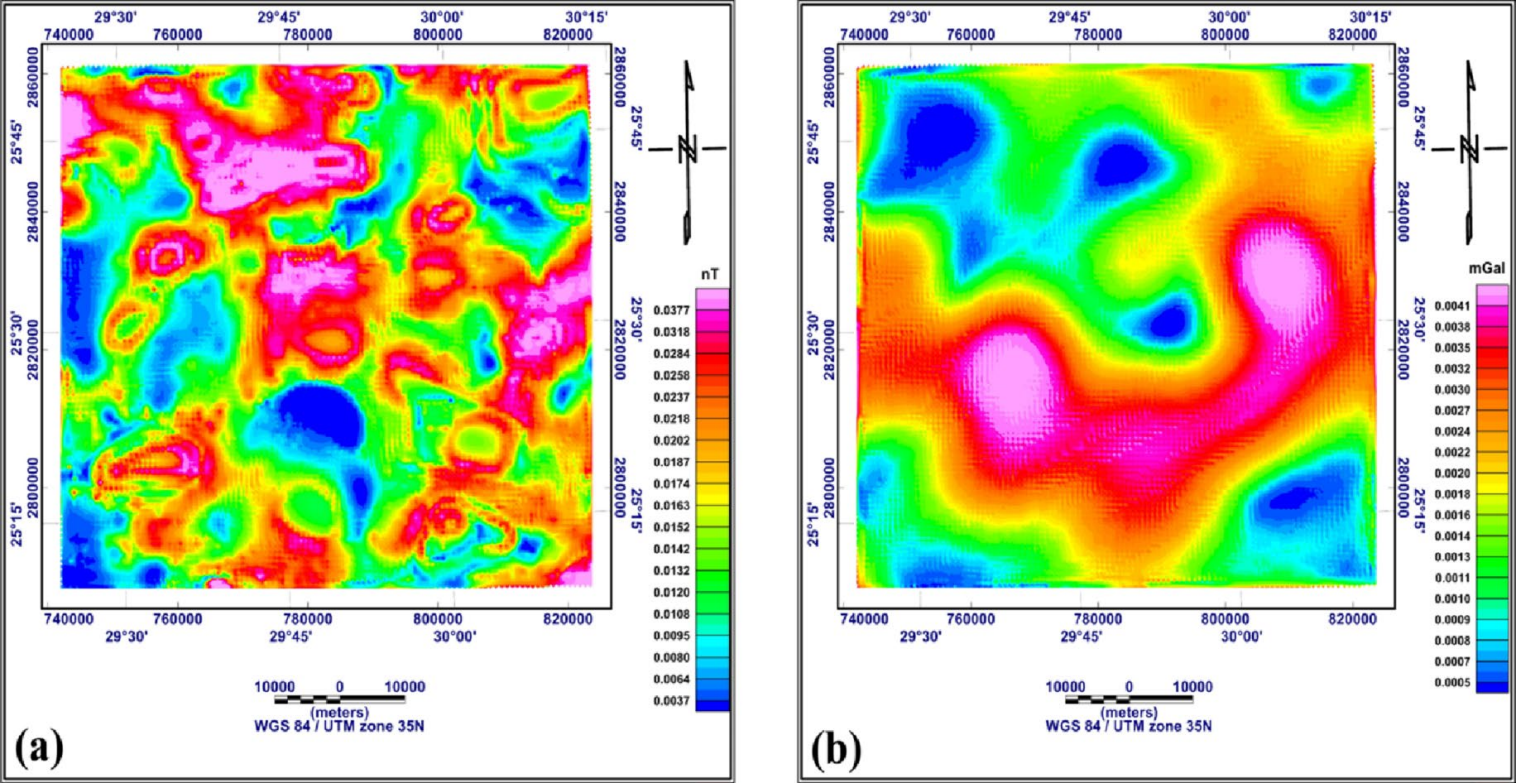
Analytical signal of the RTP aeromagnetic map (**a**) and of the Bouguer gravity map (**b**) (generated using Geosoft Oasis Montaj version 8.4 https://community.seequent.com/).

The analytical signal (AS) maps for both RTP aeromagnetic and Bouguer gravity data are shown in Fig. 10a and b, respectively. Generally, analytical signal maxima occur directly over faults and contacts. The analytical signal technique was applied to define the edges (boundaries) of bodies considered basins or uplifts in the study area. From the AS map of the RTP aeromagnetic data (Fig. 10a), the analytical signal values range from 0.0037 to 0.0377 nT. The maximum values are observed in the central and northern parts, indicating that these parts of the study area suffer from complex structures; however, the minimum values are observed in the western and southern parts, indicating simpler structures in those parts. The AS map of the Bouguer gravity data map (Fig. 10b) shows that the analytical signal values range from 0.0005 to 0.0041 mGal. low analytical signal anomalies characterize the northern part of the study area, indicating structural simplicity. However, the southern and central regions show strong AS anomalies, suggesting complex structures.

### Quantitative interpretation

The quantitative interpretation in the present study focused on 2D gravity and magnetic modelling using the GM-SYS software. From the modelling results, the top surface of the Nubian sandstone layer and the basement surface were well defined continue throughout all models. These surfaces were marked and their depth relative to the main sea level were exported as XYZ data files via the software. Furthermore, the top and bottom surfaces of the phosphatic rocks were also marked and exported in data XYZ files. From these files, the true thickness of the phosphatic rocks was estimated. Accordingly, (a) depth or structure contour maps were prepared for the top surface of the Nubian Sandstone and basement surface, and (b) an isopach map for phosphatic rocks. These maps were generated using Golden Surfer Software version15 ^60^.

#### 2D gravity and magnetic modelling

2D modelling began by outlining the most significant anomalies on the Bouguer gravity and RTP aeromagnetic maps. Such anomalies are corresponding to the predominant subsurface geologic features. About five profiles along definite lines crossing the main anomalies and extending in different directions to cover most of the study area. The technical steps of the modelling through GM-SYS of Oasis Montaj version 8.4 were as follows:

1. Importing the data files, which include the coordinates and gravity or RTP aeromagnetic values from the gridded maps. The data was in XYZ format; X is the longitude, Y is the latitude, and Z is the Bouguer gravity or RTP aeromagnetic values in mGal and nT, respectively. The XY long/lat coordinates were converted to the UTM system using the UTM projection, Datum WGS 84, ellipsoid WGS 84, and Zone 35 N.
2. Gridding data using Oasis Montaj, which provides seven different gridding algorithms to produce a grid. The gridded data is used as input to create a contour map. This map represents the aerial distribution of both Bouguer and RTP aeromagnetic anomalies, which reflect the subsurface geologic features and structures.
3. forward modelling began by entering initial parameters of the subsurface sequence, including density and magnetic susceptibility, as well as depths to the boundaries separating the expected subsurface layers or rock formations. Based on the entered initial parameters the program calculates the theoretical Bouguer gravity and RTP aeromagnetic values. After performing the calculation, the calculated values are compared to the observed values. various adjustments across several iterations were made to the initial parameters to achieve the best fit between the observed and the calculated values. The adjustments and iterations continued until low RMS values and logical subsurface model were obtained.

##### Initial parameters for the 2D modelling

**Fig. 11:**
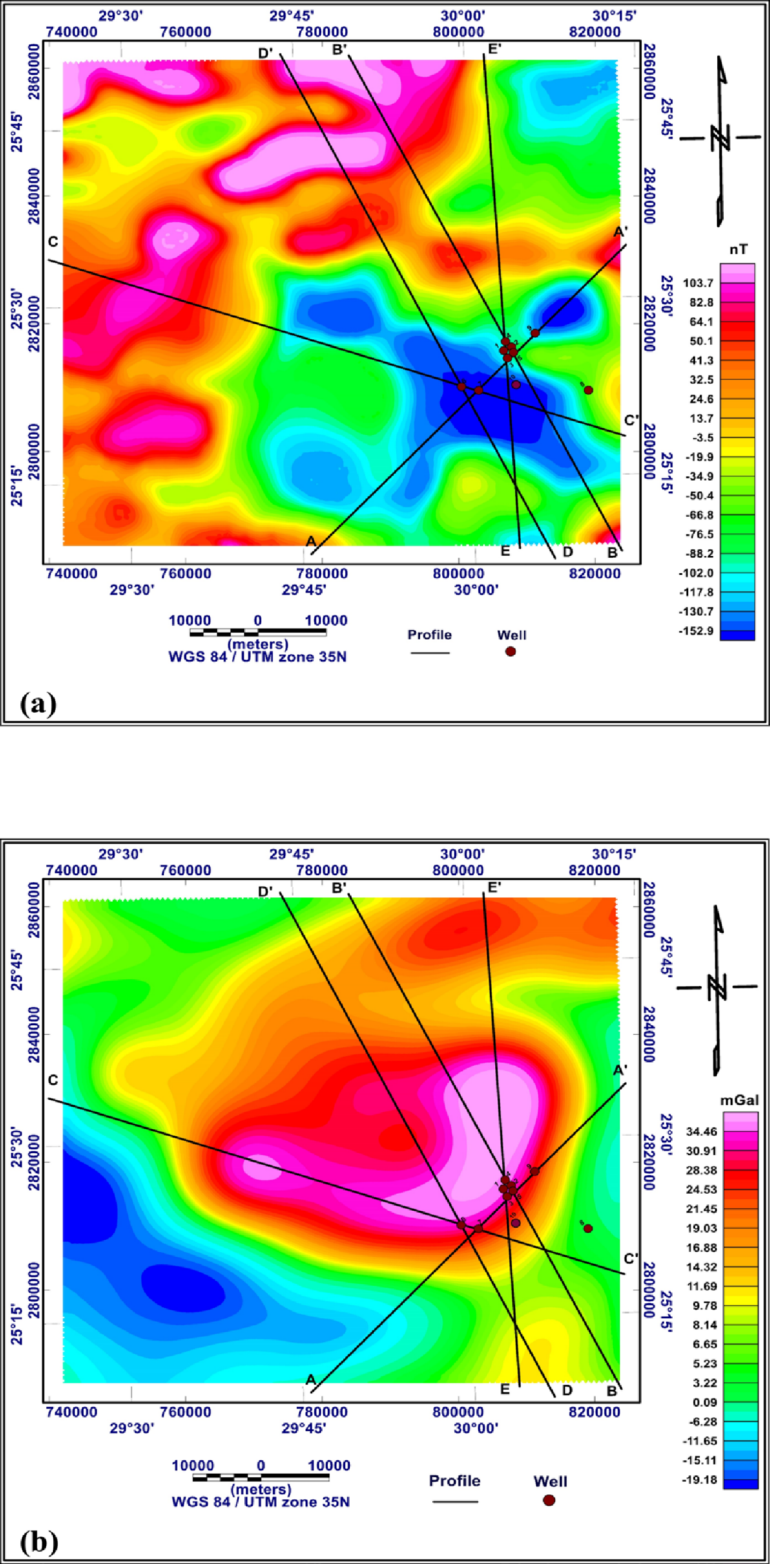
The five profiles which are selected for 2D gravity and magnetic modeling along the RTP aeromagnetic map (**a**) and the Bouguer gravity map (**b**) (generated using Geosoft Oasis Montaj version 8.4 https://community.seequent.com/).

For 2D gravity modelling, the initial parameters were the expected subsurface sedimentary sequences over the basement rocks. The expected subsurface sequence was collected from the published literature and the deep wells in the study area. For example, the Malab El-kheil well with depth to the basement rocks of 598 below sea level and the Ph2 well with a depth to the basement rocks of 755 m below sea level (Wells No. 8 and 10 respectively, in Fig. 11). The wells which not reached to the basement rocks such as those obtained from phosphate Egypt company, the depth to the basement was extrapolated based on the published work such as ^61^. The sequence over the basement rocks is started by Nubian sandstone (Nubian formation) with thickness more than 300 m ^62,63^, followed by variegated shale (Quseir formation) with thickness ranging from 100 to 225 m ^64^, followed by Duwi formation which composed from phosphatic rocks and black shale with thickness varying from 20 to 30 m, the Duwi formation is followed by Dakhla shale (Dakhla formation) with thickness ranging from 100 to 135 m, followed by Limestone belonging to (KurKur formation) with thickness varying from 25 to 100 m ^62,63^. The average density of the mentioned rock types was obtained from the literatures, such as ^65–68^, and textbooks 52,69–71. Accordingly, the average densities of the subsurface sequence in the study area can be summarized as follows: Limestone (2.5–2.7 g/cm³), Dakhla shale (1.77–2.6 g/cm³), Phosphatic rocks (1.52–2.97 g/cm³), Variegated shale (2.63–2.65 g/cm³), Nubian sandstone (2.65–2.67 g/cm³) and Basement rocks with density ranging from 2.52 to 3.45 g/cm³.

For 2D magnetic modelling, the initial parameters were the magnetic susceptibility of the basement rocks and the overlying sedimentary cover. The magnetic susceptibility of the basement rocks in and around the study area ranges from 0.001 to 0.0058 cgs units 72, equivalent to 0.013–0.073 in SI units and the magnetic susceptibility of the sedimentary cover is negligible.

The five profiles selected for 2D modelling are shown on both the RTP aeromagnetic and Bouguer maps (Fig. 11a and b). The profiles were nominated, A-À, B-B `, C-C `, D-D ` and E-E `. The Profile A-À is with length 64.006 km, Profile B-B ` is with length 85.041 km, Profile C-C ` is with length 85.714 km, Profile D-D ` is with length 84.936 km, and Profile E-E ` is with length 776.1624 km.

**Fig. 12:**
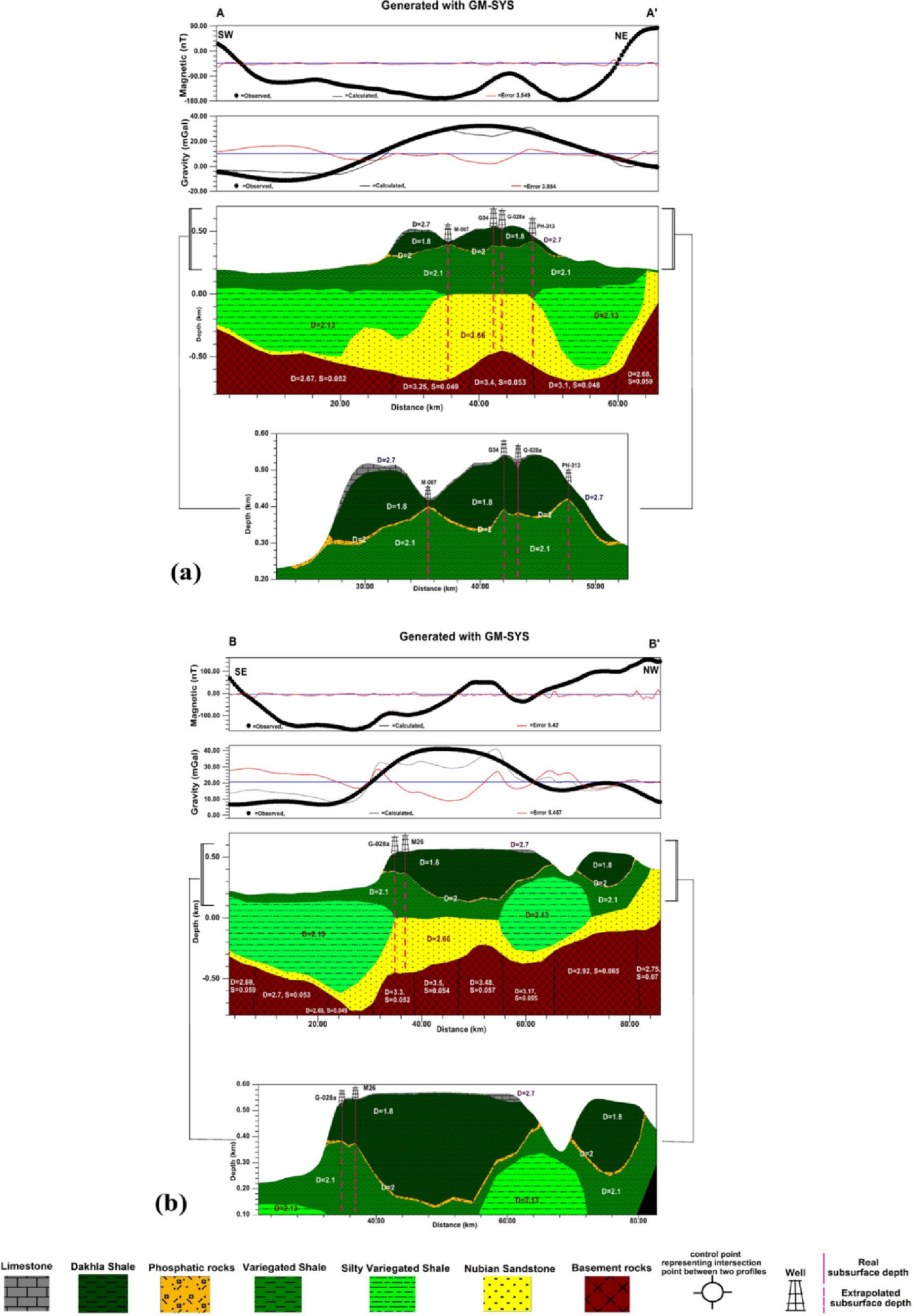
2D Gravity and magnetic modelling along A-A` profile (**a**), 2D Gravity and magnetic modelling along B-B` profile (**b**), where D= Density of rock (g/cc), S= Magnetic susceptibility (SI), Black points represent the observed data, while black curves represent the calculated responses from the model (generated using Geosoft Oasis Montaj version 8.4 https://community.seequent.com/).

**Fig. 13:**
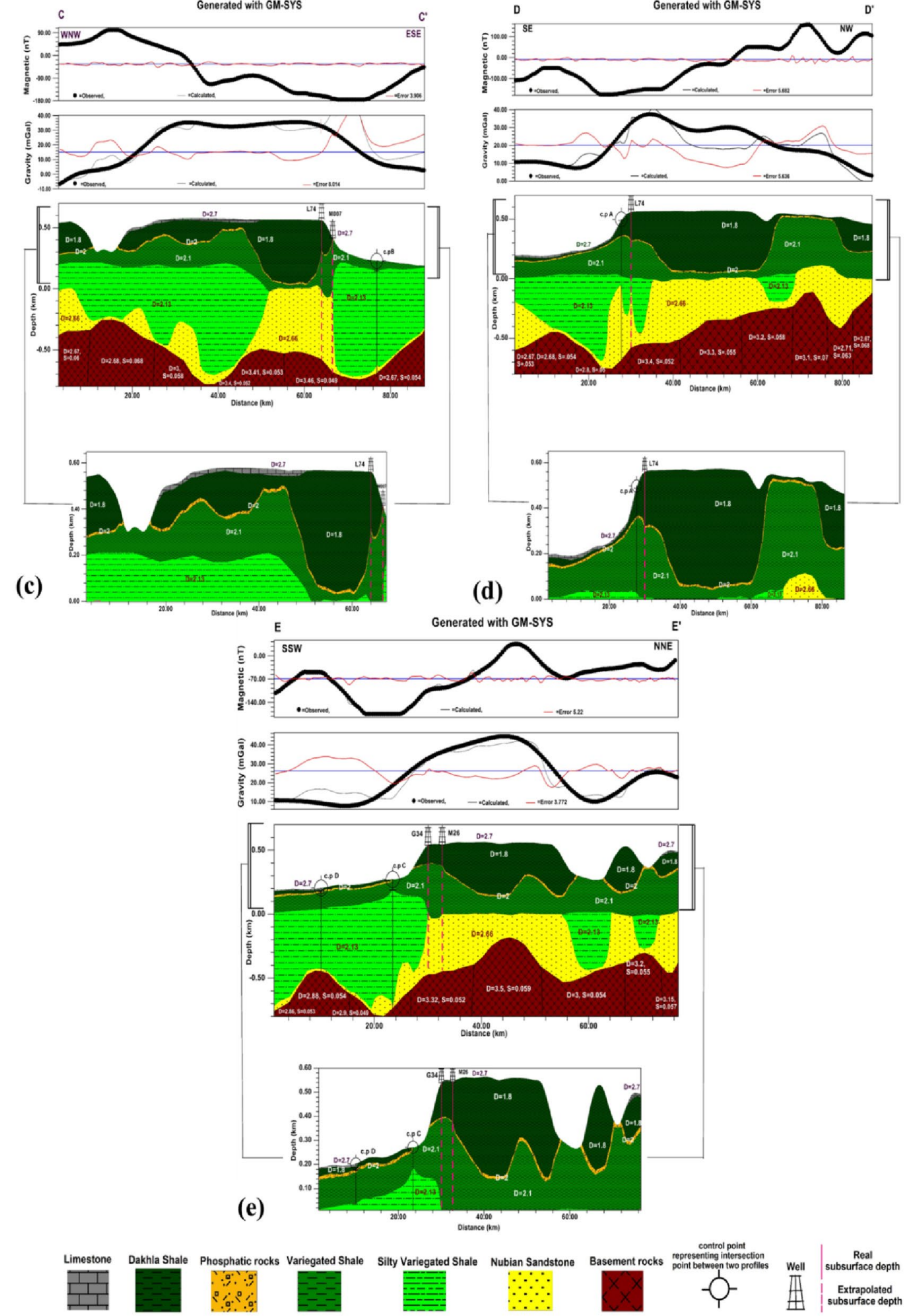
2D Gravity and magnetic modelling along C-C` profile (**c**), 2D Gravity and magnetic modelling along D-D` profile (**d**), 2D Gravity and magnetic modelling along E-E` profile (**e**), where D= Density of rock (g/cc), S= Magnetic susceptibility (SI) Black points represent the observed data, while black curves represent the calculated responses from the model (generated using Geosoft Oasis Montaj version 8.4 https://community.seequent.com/).

Figures 12 and 13 show the 2D gravity and magnetic modeling and the obtained subsurface geologic models. The best fit between the calculated and observed gravity and magnetic values obtained by following the RMS values in GM-SYS of Oasis Montaj version 8.4. The accepted RMS values for all modeled profiles ranged from 3.549%. to 8.014%. This range of RMS values falls within the commonly accepted range, generally below 15%. The acceptance of the models, aside from the RMS values, was based on the correspondence of the obtained models with the real subsurface sequence in the study area. Furthermore, the obtained models were constrained by subsurface data from deep wells drilled in the study area.

From the interpretation of five profiles and the corresponding 2D gravity and magnetic modelling, A-A`, B-B`, C-C`, D-D`, and E-E` Which extend SW to NE, SE to NW, WNW to ESE, SE to NW, and SSW to NNE, respectively, and pass through the available wells in the study area, the subsurface sequence can be summarized as follows:

- The basement rocks are situated at depths range from 138 to 798 m below sea level, with average density ranges from 2.6 to 3.5 g/cm³ and magnetic susceptibilities ranging from 0.048 to 0.07 SI units. These remarkable variations in both density and magnetic susceptibility indicate lateral variations in the mineral composition of the basement rocks, which may also reflect lithologic contacts between different basement rock types.
- The Nubian sandstone layer, which is defined as the Nubian Sandstone Formation in literatures, has an average thickness ranges from 90 to 525.53 m and an average density of 2.66 g/cm³. This significant variation in thickness may indicate that this formation is strongly affected by faulting.
- The variegated shale layer, which is defined as the Quseir Formation in literature, has an average thickness of 58.76 to 374.13 m and an average density of 2.1 g/cm³. This formation appeared in the models as continuous shale layer and silty shale lenses, with a density of 2.13 g/cm³ in some places. The lenses have average thickness of 498.86 to 765.524 m. These high thickness values of may be due to the silty shale lenses deposited in deep lagoon or lake environments, with density of 2.13 g/cm³.
- The Dakhla shale layer, which is defined as the Dakhla Formation in the literature, is observed with an average density 1.8 g/cm³ as a continuous layer along the D-D` model, with thickness ranging from 30.61 to 510.20 m. Whereas, they appeared as separated blocks along A-A`, B-B`, C-C`, and E-E` models with average thickness varies from 201.5 to 396.15 m. The blocks are predominant at the parts which may be affected by faulting and weathering.
- The limestone layer, which is defined as the Kurkur Formation in the literature, with average density of 2.7 g/cm³ appears to cap the sedimentary sequence; hence, its actual thickness is not estimated.
- The main objective of the present study was to examine the mode of occurrence of the phosphatic rocks; the parts of the models that contain them are zoomed out and plotted below the models. The phosphatic rocks are defined as Duwi Formation in the literatures. From the zoom out parts, the phosphatic rocks with average density 2 g/cm³ are observed as a continuous layer along A-A` and D-D` models with average thickness ranges from 1.75 to 19.9 m and as a disconnected layer along B-B`, C-C`, and E-E` models with average thickness varies from 1.9 to 23.46 m. These great thickness variations and disconnection in the phosphatic rocks may be due to faults that are predominant in the study area.
- Lateral thickness variation in the subsurface sequence from the Nubian sandstone at the bottom to the Kurkur Formation at the top may indicate that the entire sequence is affected by structures originating in the basement rocks and extending upward into the sedimentary cover.

##### Depth or structure contour map of the basement surface

**Fig. 14:**
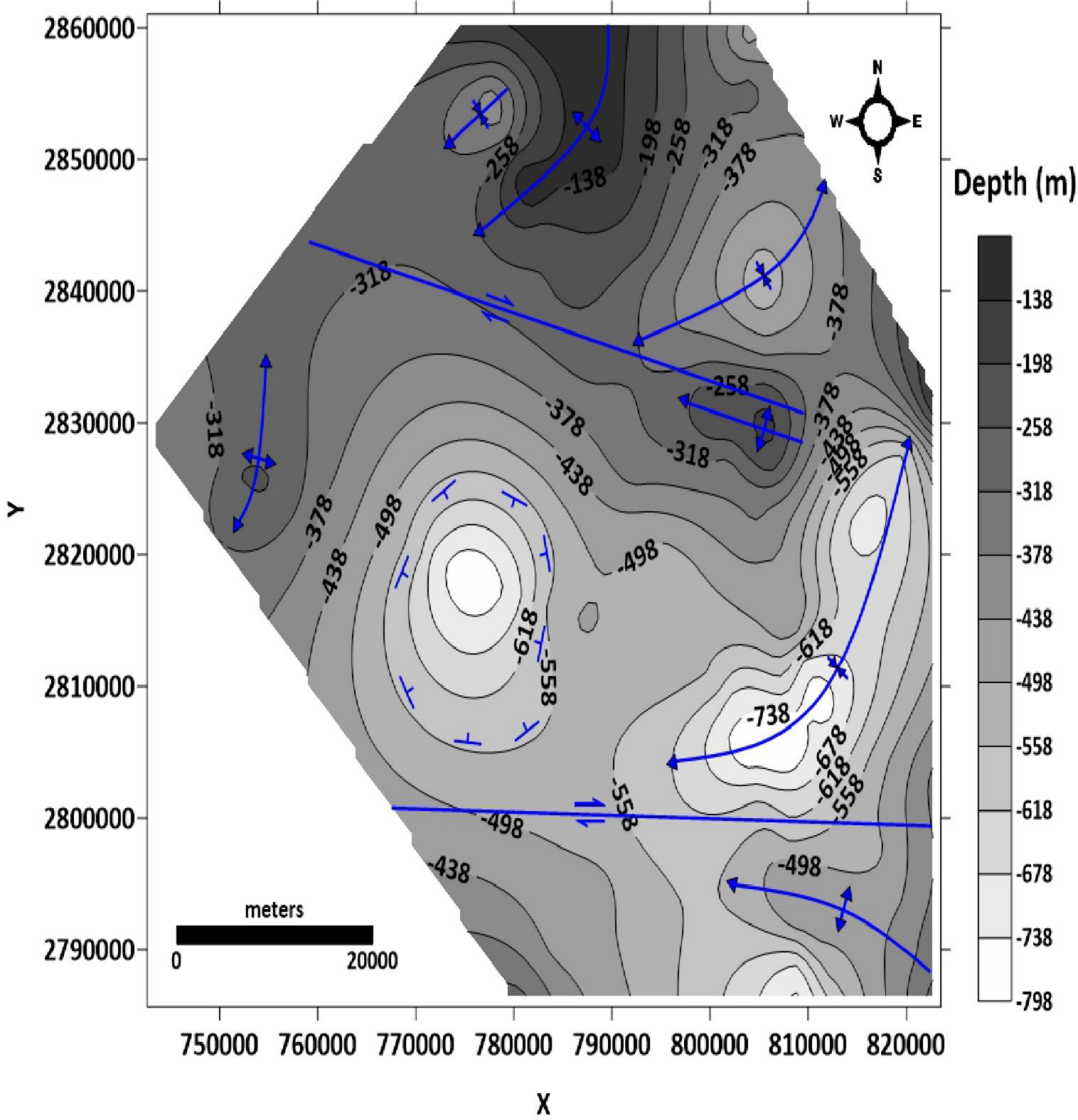
Structure contour map of the basement surface (generated using Golden Surfer Software version15).

From the prepared map of the basement surface in the study area (Fig. 14), the main structures are two syncline folds located at the northern and eastern parts of the study area. These syncline folds are asymmetrical, double-plunging, with an axis extending NE-SW direction. Another symmetrical plunging syncline fold with an axis extending in the NE-SW is observed at the northwest part of the study area. At the western part, the contours show a basin structure. There are four anticlines located in the northern, eastern, western, and southern parts of the study area. The anticline fold in the northern part is asymmetrical, plunging with an axis extending NE-SW direction. The anticline fold in the eastern part is symmetrical plunging with an axis extending WNW-ESE. The anticline fold in the southeastern part is asymmetrical plunging with an axis extending WNW-ESE. The anticline fold in the western part is symmetrical, plunging with an axis extending NNE-SSW. Two dextral strike-slip faults are also observed; the first one is in the northern part of the study area, extending WNW-ESE, and the other is in the southern part, extending W-E.

##### Depth or structure contour map of the top Nubian sandstone layer

**Fig. 15:**
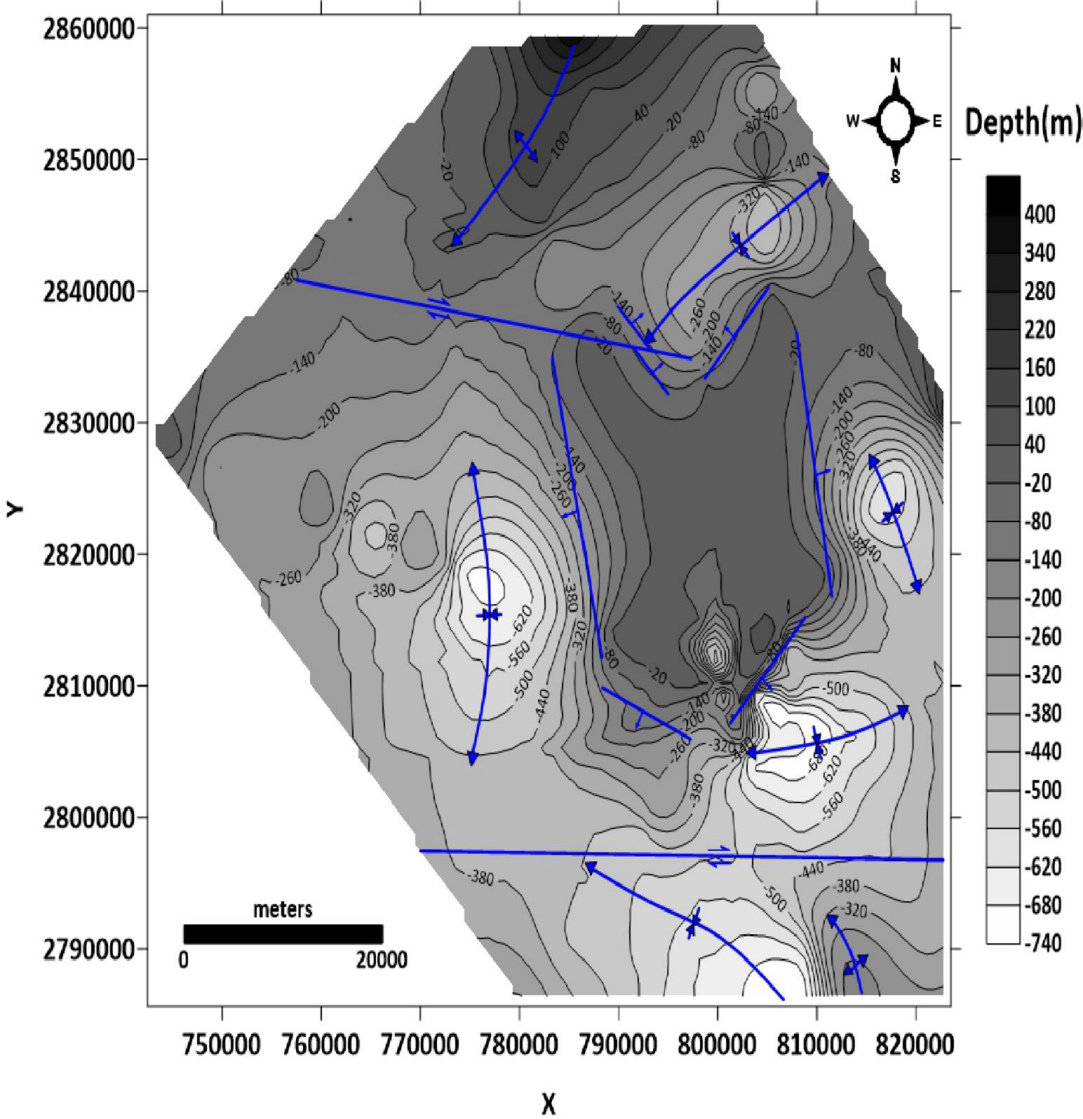
Structure contour map of the top Nubian sandstone surface (generated using Golden Surfer Software version 15).

From the structure contour map of the top Nubian sand surface in the study area (Fig. 15), the main structures are five synclines located in the northern, eastern, western, southeastern, and southern parts of the study area. The syncline fold at the northern part is asymmetrical double-plunging with an axis extending NE-SW. The syncline fold at the western part of the study is asymmetrical double-plunging with an axis extending NNW-SSE. The syncline fold at the eastern part is symmetrical double-plunging with an axis extending NNW-SSE. The syncline fold at the southeastern part is asymmetrical double-plunging with an axis extending W-E. The syncline fold at the southern part of the study area is asymmetrical plunging with an axis extending WNW-ESE. Two anticline folds are defined in the northern and southern parts of the study area. The anticline fold at the northern part is asymmetrical plunging with an axis extending NE-SW. The anticline fold at the southeastern part is asymmetrical plunging with an axis extending NW-SE. Six normal faults are observed at the middle part of the study area, bounding Abu Tartur plateau and extending NNW-SSE, NE-SW, and NW-SE. The downthrows of these faults are due to the lowlands outside of the plateau. Two dextral strike-slip faults, one of them in the northern part of the study area extending WNW-ESE direction and the other one in the southern part extending W-E direction.

##### Thickness or Isopach map of the phosphatic rocks

**Fig. 16:**
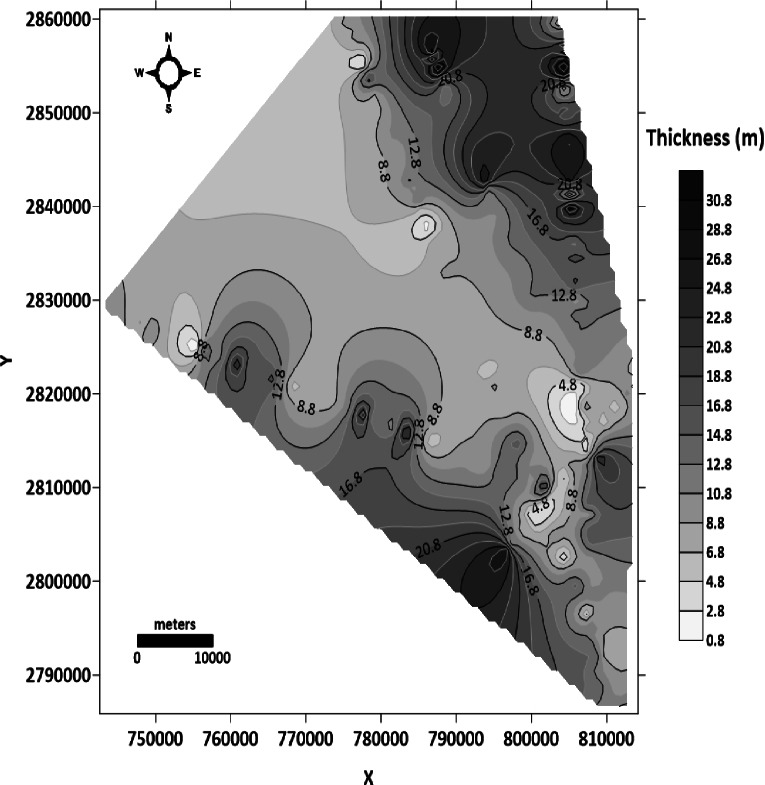
Isopach map of phosphatic rocks (generated using Golden Surfer Software version15).

The prepared thickness map of the phosphatic rock (Fig. 16) show that:

- The subsurface thickness of the phosphatic rocks ranges between 0.8 and 32 m. This great lateral variation in the thickness is related to the predominant structures.
- The maximum thickness is recorded in the northeast and southwest parts of the Abu Tartur plateau.

**Fig. 17:**
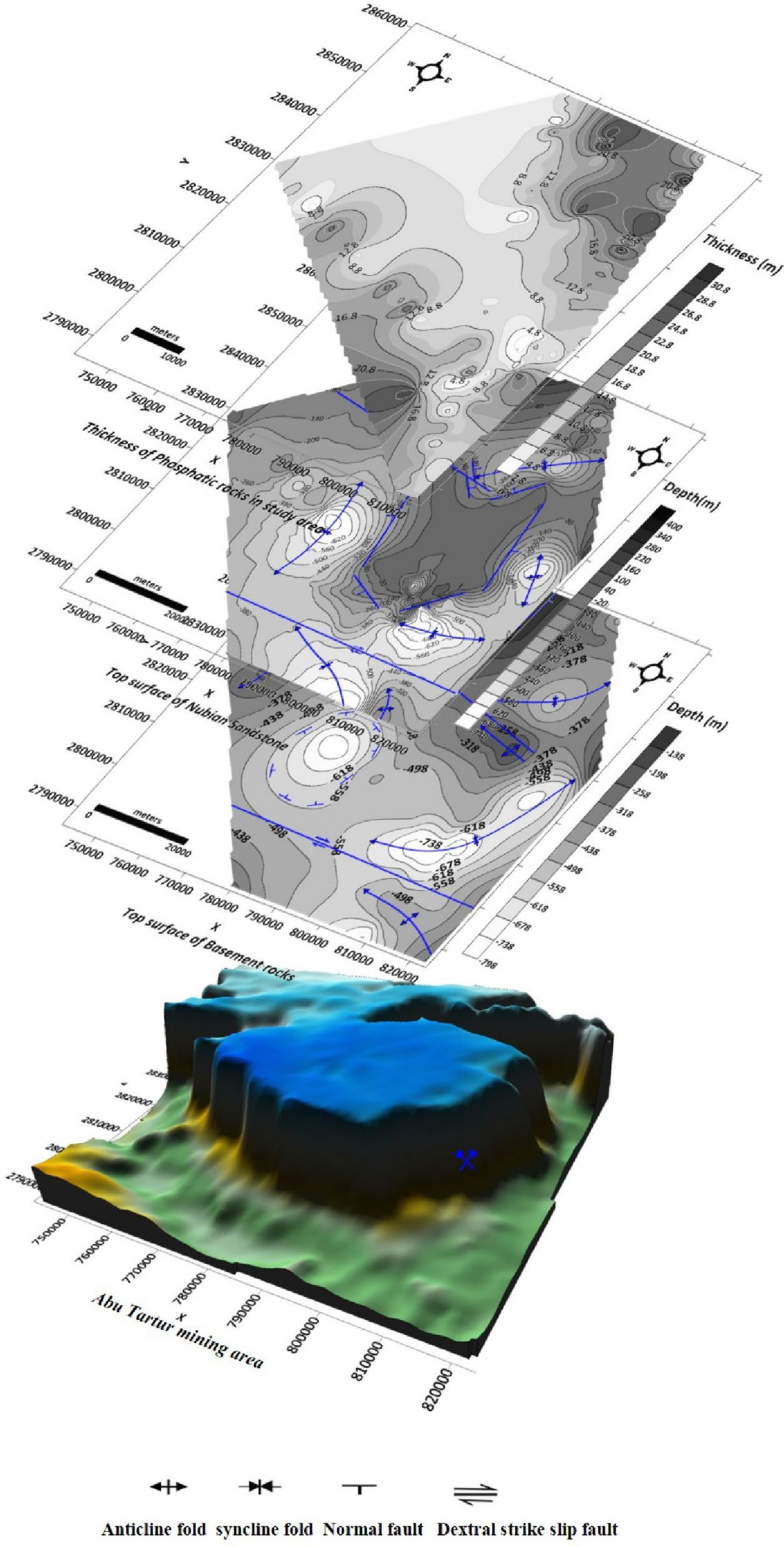
Combination of structure contour map of the basement surface, structure contour map of the top Nubian sandstone surface and thickness map of the phosphatic rocks with the 3D view of the Abu Tartur plateau (generated using Golden Surfer Software version15).

Figure 17 illustrates the combination of the structure contour map of the basement surface (Fig. 14), the structure contour map of the top Nubian sandstone surface (Fig. 15), and the thickness map of phosphatic rocks (Fig. 16). From the cumulative figure (Fig. 17), It can be noticed that the maximum thickness of the phosphatic rocks is observed at the troughs of the syncline folds, which are located northeast and southwest of Abu Tartur plateau. several faults bound the Abu Tartur plateau with downthrow outward from the plateau. So, the present location of the Abu Tartur mine was ideal for underground mining and, accordingly, not economic.

### Structure trends analysis of lineaments detected from Bouguer gravity and RTP aeromagnetic maps

The most important step in interpreting Bouguer gravity and RTP aeromagnetic data is turning them into tectonic information. Tectonic interpretation of a particular area is strongly influenced by the main structural features of the basement surface that affect the sedimentary cover above. The main tectonic trends influencing the study region are visualized in the current work using trend analyses of the gravitational and magnetic fields and their derivatives. The digitizing techniques available in Rockware Software version15 ^73^ were used to trace and measure the structural lineaments. The main structural trends prevailing in the study area can be detected as follows: N-S trend, which azimuths from 0˚to 5˚ to the west or the east, NNE trend from 5˚ to 30˚ to the east, NNW trend from 5˚to 30˚ to the west, NE trend from 30˚to 60˚ to the east, NW trend from 30˚to 60˚ to the west, ENE trend from 60˚to 85˚ to the east, WNW trend from 60˚to 85˚ to the west and W-E trend from 85˚to 90˚ to the west or east. (Table 1) summarizes the structural trends extracted from the gravity and magnetic data across different processing stages, including RTP, Bouguer, regional, residual, FVD, TDR, and AS maps.

**Table 1:** Summary of the trends detected from different maps of the study area.

Directions	Corresponding maps			Domain Type			
	1st Trends	2nd Trends	3rd Trends	Major Trends		Minor Trends	
				Regional RTP	Regional Bouguer	Local RTP	Local Bouguer
W-E	AS gravity, FVD RTP, FVD gravity, residual RTP, residual gravity, TDR RTP, TDR gravity	RTP, regional gravity	AS RTP, Bouguer gravity, regional RTP	√	√	√	√
ENE	AS RTP, RTP, TDR RTP	FVD RTP, FVD gravity, regional RTP, regional gravity, residual RTP, residual gravity	Bouguer gravity, TDR gravity	√	√	√	√
NNE	Bouguer gravity, FVD RTP	AS gravity, FVD gravity, regional gravity, TDR RTP	Regional RTP, Residual RTP, TDR gravity	√	√	√	×
NW	Regional RTP	AS RTP, Bouguer gravity, FVD gravity, regional gravity	Residual RTP	√	√	√	×
NNW	Regional gravity	AS RTP, Bouguer gravity	FVD gravity, RTP, regional RTP, TDR RTP	√	√	×	×
WNW	Regional gravity	AS RTP, RTP, regional RTP, TDR gravity	Bouguer gravity, FVD RTP, FVD gravity, residual gravity	√	√	×	√
NE	–	Bouguer gravity, regional gravity	AS RTP, AS gravity, RTP, regional RTP	√	√	×	×

**Fig. 18:**
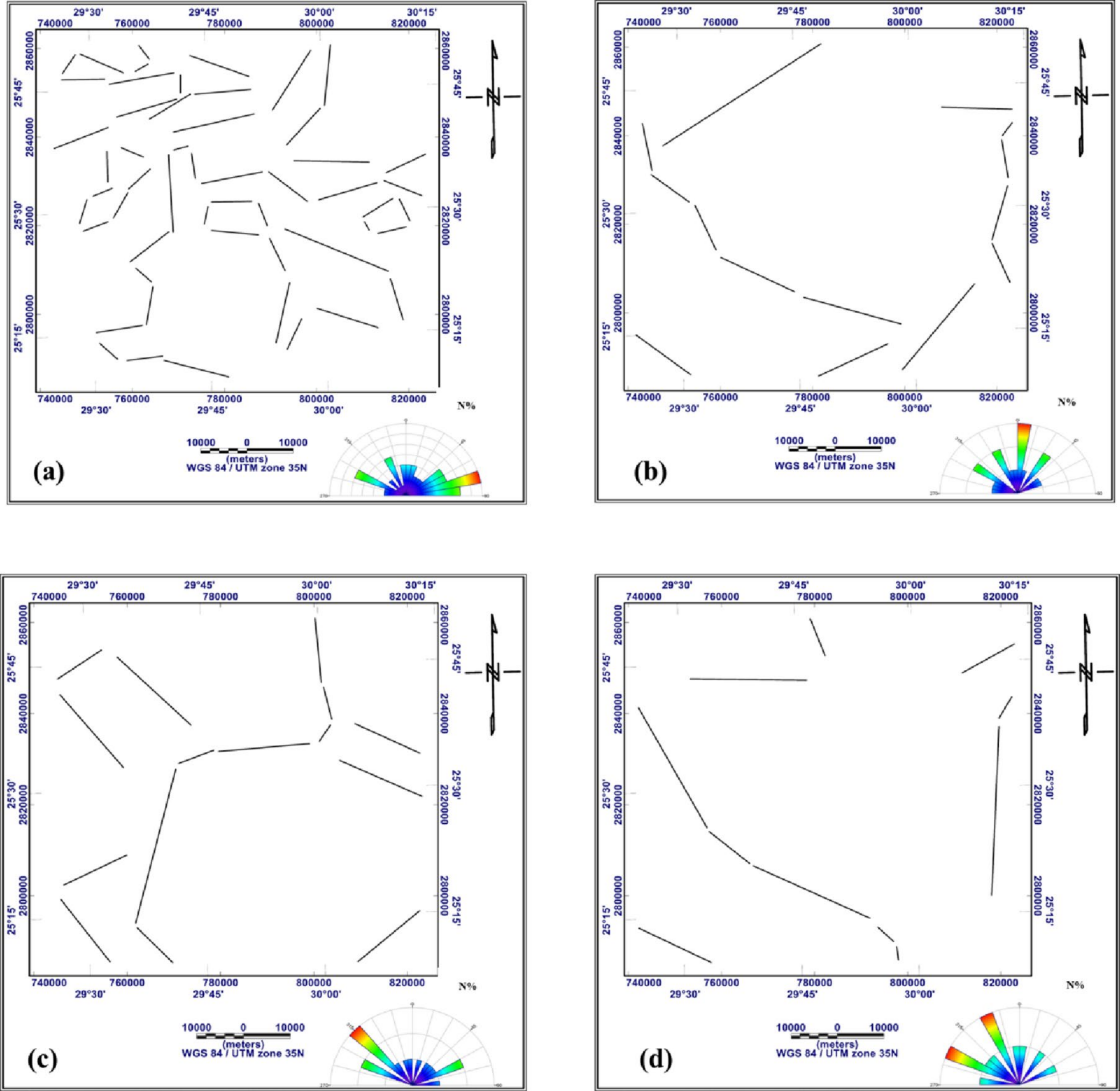
Structure lineaments traced from: the residual map of RTP aeromagnetic data (**a**), the residual map of Bouguer gravity data (**b**), the FVD map of RTP aeromagnetic data (**c**), the FVD map of Bouguer gravity data (**d**), the TDR map of RTP aeromagnetic data (**e**) and the TDR map of Bouguer gravity data (**f**) (generated using Rockware Software version15 https://www.rockware.com/).

**Fig. 19:**
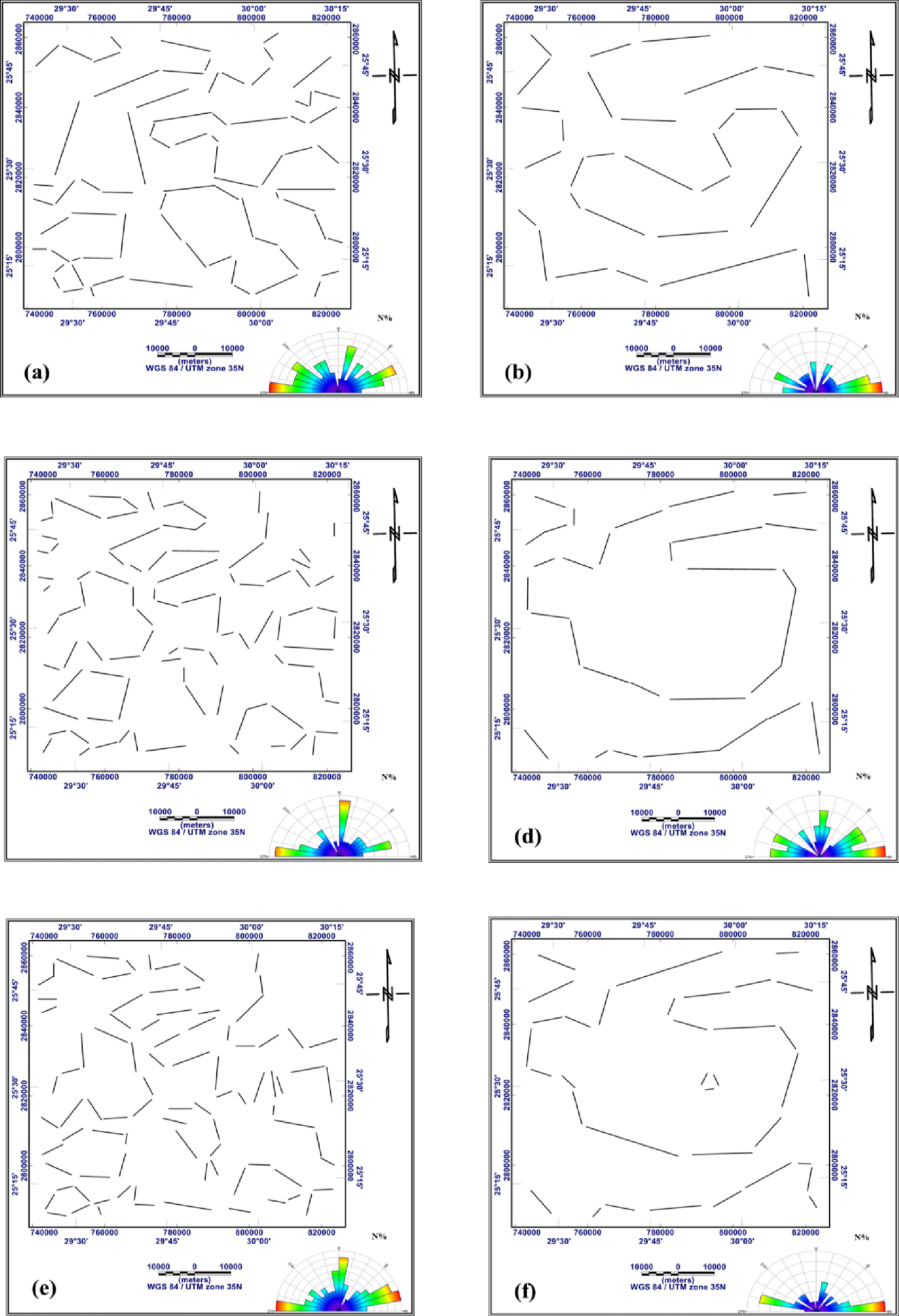
Structure lineaments traced from the AS map of RTP aeromagnetic data (**a**) and from the AS map of Bouguer gravity data (**b**) (generated using Rockware Software version15 https://www.rockware.com).

**Fig. 20:**
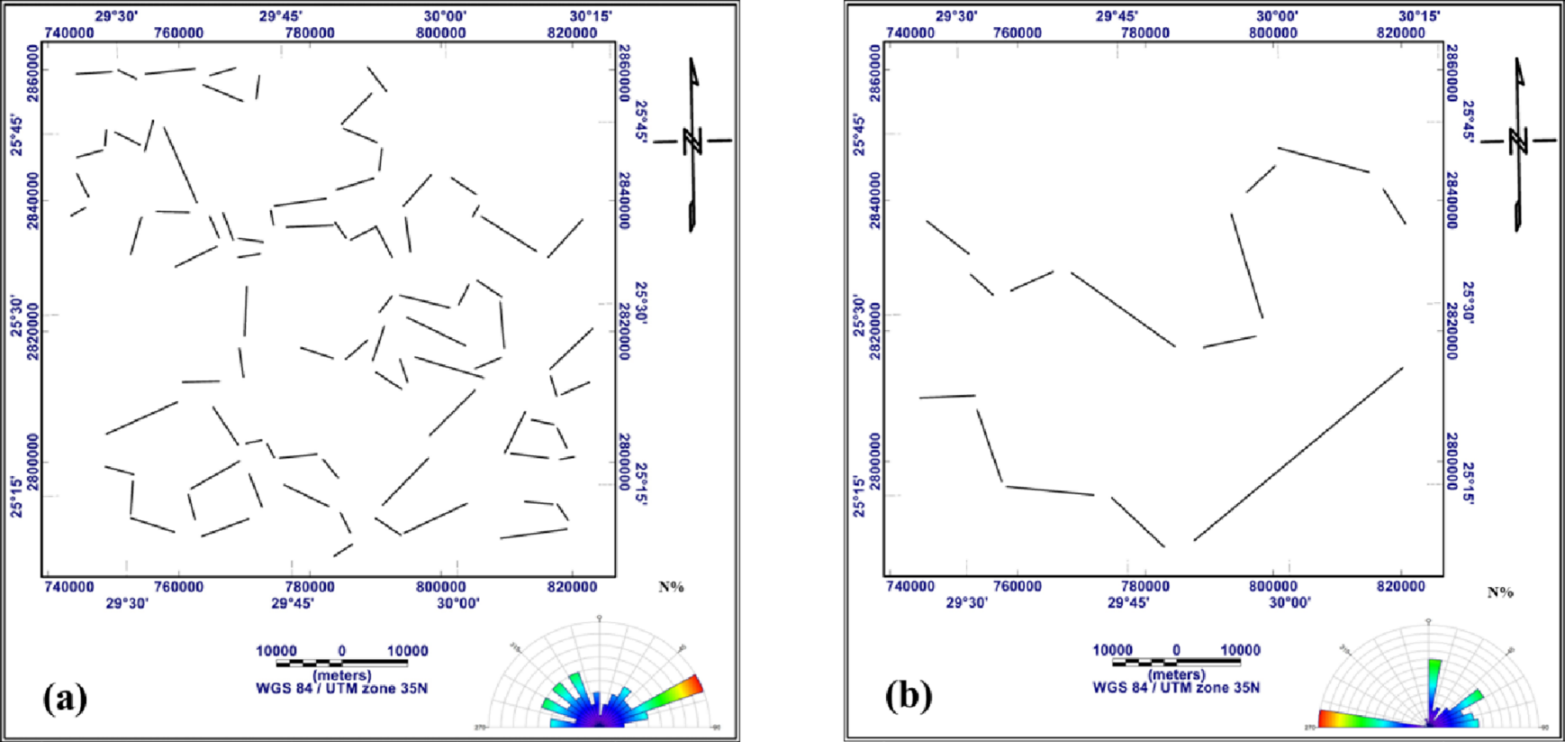
Structure lineaments traced from: the RTP aeromagnetic map (**a**), the Bouguer gravity map (**b**), the regional map of RTP aeromagnetic data (**c**) and the regional map of Bouguer gravity data (**d**) (generated using Rockware Software version 15 https://www.rockware.com/).

- **W-E Trend**

This is the most prominent trend recorded in the study area, it was found as first order in residual RTP, residual gravity, FVD RTP, FVD gravity, TDR RTP, TDR gravity and AS gravity (Figs. 18a, b, c, d, e and f and 19b), recorded as second order in RTP and regional gravity (Fig. 20a and d), and as third order in Bouguer gravity, regional RTP, and AS RTP (Figs. 20b and c and 19a). This trend has been recorded by several recent studies 56,74–76.

b) **ENE-WSW Trend**

This trend has been verified through several recent studies 77,78. In RTP, TDR RTP, and AS RTP (Figs. 20a, 18e and 19a) this trend is recorded as first order, observed as second order in regional RTP, regional gravity, residual RTP, and residual gravity, FVD RTP, and FVD gravity (Figs. 20c and d and 18a, b, c and d), and in the Bouguer gravity and TDR gravity (Figs. 20b and 18f), this trend is characterized as third order.

c) **NNE-SSW Trend**

This trend is observed as the first order in Bouguer gravity and FVD RTP (Figs. 20b and 18f), as second order in regional gravity, FVD gravity, TDR RTP, and AS gravity (Figs. 20d, 18d and e and 19b), and as third order in regional RTP, residual RTP and TDR gravity (Figs. 20c and 18a and f). This trend has been identified by many authors 79–81.

d) **NW-SE Trend**

This trend manifests as a first order in the regional RTP map (Fig. 20c), as a second order in Bouguer gravity, regional gravity, FVD gravity, and AS RTP (18b, 18d, 19d and 20a), and as a third order in residual RTP (Fig. 18a). This trend has been recorded by ^67,76,82,83^.

e) **NNW-SSE Trend**

On the regional gravity (Fig. 20d), this trend appears as first order. Second order signature in Bouguer gravity and AS RTP (Figs. 20b and 19a), whereas third order expression in RTP, Regional RTP, FVD gravity and TDR RTP (Figs. 20a and c and 18d and e) This trend has been confirmed by several studies 74,84,85.

f) **WNW-ESE Trend**

This trend is delineated as the first order in regional gravity (Fig. 20d), recorded as the second order in RTP, regional RTP, TDR gravity, and AS RTP (Figs. 20a and c, 18e and 19a), and in the Bouguer gravity, residual gravity, FVD RTP, and FVD gravity (Figs. 20b and 18b, c and d), this trend is characterized as the third order. This trend has been identified by ^67,74,86,87^.

g) **NE-SE Trend**.

This trend has been verified through 74,75,84. This trend is observed as second order in Bouguer gravity and regional gravity (Fig. 20b and d). It has appeared as a third order in RTP, regional RTP, AS RTP, and AS gravity (Figs. 20a and c and 19a and b).

## Conclusions

This study applied an integrated geophysical approach, combining aeromagnetic (RTP) and Bouguer gravity data, to characterize the subsurface structural framework of Abu Tartur Phosphate Mine (ATPM). The results provide critical insights into the geological controls on phosphate distribution, which are essential for optimal mine planning and sustainable resource utilization.

Qualitative interpretation of the gravity and magnetic data revealed several dominant structural trends in the region, including W-E, ENE, NNE, NW, NNW, WNW, and NE directions. These trends are associated with both deep seated basement structures and shallower features observable near the surface. Quantitative analysis through 2D modelling confirmed the presence of complex subsurface structures, mainly comprising normal and strike-slip faults, as well as plunging and double-plunging folds with axes trending NE-SW, NNW-SSE, and NW-SE.

The remarkable feature of this study is the significant lateral variation in the thickness of the phosphatic rocks, ranging from 0.8 to 32 m (based on the 2D modelling). The thinnest layers were identified in the currently exploited central plateau area, which likely contributed to the subeconomic performance and eventual closure of the underground mine. Conversely, the thickest phosphate deposits were found at the troughs of synclinal folds located northeast and southwest of the plateau zones that offer substantially greater mining potential.

These results clearly demonstrate that the current ATPM site was suboptimal for underground mining and that better-informed geophysical analysis could have prevented the misallocation of resources. The integration of gravity and magnetic data, supported by borehole data and geological modelling, proved essential for delineating structurally controlled phosphate rich zones. This approach not only enhances the economic feasibility of future mining operations at ATPM but also provides a replicable framework for mineral exploration in structurally complex terrains elsewhere.

Ultimately, this research underscores the importance of multidisciplinary geophysical characterization to guide resource extraction, reduce risk and supporting long-term sustainability in the mining sector.

## Data Availability

No additional data to declare. All data generated or analyzed during this study are included within this manuscript.
